# A base editing platform for the correction of cancer driver mutations unmasks conserved p53 transcription programs

**DOI:** 10.1186/s13059-025-03667-7

**Published:** 2025-07-22

**Authors:** Pascal Wang, Rituparno Sen, Frank Buchholz, Shady Sayed

**Affiliations:** 1 Medical Systems Biology, Faculty of Medicine Carl Gustav Carus, TU Dresden, Dresden, Germany; 2 National Center for Tumor Diseases (NCT), NCT/UCC Dresden, a partnership between DKFZ, Faculty of Medicine and University Hospital Carl Gustav Carus, TUD Dresden University of Technology, and Helmholtz-Zentrum Dresden-Rossendorf (HZDR), Dresden, Germany; 3 German Cancer Consortium (DKTK), Dresden, Germany

**Keywords:** Cancer, Base editing, P53, Driver mutation, Transcriptomics, SMAD4, CRISPR

## Abstract

**Background:**

Understanding the role of cancer hotspot mutations is essential for unraveling mechanisms of tumorigenesis and identifying therapeutic vulnerabilities. Correcting cancer mutations with base editing is a novel, yet promising approach for investigating the biology of driver mutations.

**Results:**

Here, we present a versatile platform to investigate the functional impact of cancer hotspot mutations through adenine base editing in combination with transcriptomic profiling. Using this approach, we correct *TP53* hotspot mutations in cancer cell lines derived from diverse tissues, followed by mRNA sequencing to evaluate transcriptional changes. Remarkably, correcting these mutations not only reveals the dependency on mutant allele expression but also restores highly conserved tumor-suppressive transcriptional programs, irrespective of tissue origin or co-occurring mutations, highlighting a shared p53-dependent regulatory network. Our findings demonstrate the utility of this base editing platform to systematically interrogate the functional consequences of cancer-associated mutations and their downstream effects on gene expression.

**Conclusions:**

This work establishes a robust framework for studying the transcriptional dynamics of cancer hotspot mutations and sheds light on the conserved biological processes reinstated by p53 correction, offering potential avenues for future targeted therapies.

**Supplementary Information:**

The online version contains supplementary material available at 10.1186/s13059-025-03667-7.

## Background

Cancer remains one of the most challenging diseases to treat, owing to its complex, adaptive, and multifaceted nature [1, 2]. A hallmark of cancer cells is the presence of cancer driver mutations—genetic alterations that provide a selective growth advantage, driving tumor initiation and progression [3, 4]. These mutations are central to the malignant transformation of normal cells and typically occur alongside additional driver mutations and numerous benign “passenger” mutations [5]. Current treatment strategies, including chemotherapy [6, 7], radiation [8], targeted therapies [9], and immunotherapies [10], aim to exploit vulnerabilities conferred by these driver mutations. Despite these advancements, understanding the functional consequences of driver mutations and how they interact with co-occurring mutations remains a significant barrier to improving therapeutic outcomes [5, 11]. Ideally, investigations should assess cancer driver mutations within their native genomic context as well as in the setting of naturally developed tumors [11–13].

Among the most well-defined driver mutations are the recurrent hotspot mutations in the tumor suppressor gene *TP53*, often referred to as the “guardian of the genome” [14]. *TP53* mutations, occurring at hotspots such as R175, G245, R248, R273, and R282, are among the most common genetic alterations in human cancers [15–17], collectively present in 5–10% of all cancer samples tested [16, 18, 19]. These mutations disrupt p53’s role as a transcriptional regulator of apoptosis, cell cycle control, and senescence, contributing to tumorigenesis [18]. Interestingly, the same *TP53* hotspot mutations are observed across diverse cancer types, typically alongside a wide spectrum of other driver mutations [20, 21], suggesting that *TP53* mutations cooperate with many different cancer drivers. However, while pioneering studies had shown that overexpression of wt p53 in cancer null lines induces growth disadvantages [22, 23], it remains unclear whether repairing the same hotspot mutations on their natural locus can exert the same consistent effects across different tumor contexts or if their functional consequences depend on the co-occurring mutational landscape [24–26]. This fundamental question poses a significant challenge to the development of targeted therapies, as understanding whether the effects of *TP53* mutations are tumor-agnostic or context-specific could shape therapeutic strategies [17, 27, 28].

Addressing this question has proven difficult due to the inherent challenges of studying the effects of individual mutations in the context of naturally occurring tumor genomes [29, 30]. Traditional models, such as using transgenes or engineered knock-in mutations, have provided foundational insights but also come with significant limitations [31, 32]. Transgenic models often involve random integration of the mutation under an artificial or non-native promoter, leading to non-physiological expression as well as bypassing endogenous enhancers, silencers, and feedback loops, all while bearing the risk of insertional mutagenesis [33]. Meanwhile, knock-in models examine a specific mutation at the endogenous locus but often fail to fully capture the dynamic and multifaceted nature of tumor evolution in addition to being costly, time-consuming, and low-throughput [34, 35]. Recent advances in base-editing technologies, such as adenine base editors (ABEs) [36], offer a precise, efficient, and tunable method for correcting driver mutations in their native genomic context [37–40]. This approach enables direct interrogation of the functional consequences of specific mutations in cancer cells, providing a powerful tool for studying driver mutations.

In this manuscript, we expand on the correction of cancer driver mutations in a single cell line [37], and explore the correction of five cancer hotspot mutations across six distinct cell lines and integrate adenine base editing with transcriptomic profiling to assess the functional impact of correcting two different *TP53* mutations. Our findings reveal a highly conserved transcriptional p53 response following mutation correction, suggesting that the effects of *TP53* hotspot mutations are largely independent of tumor origin and co-occurring mutations. We also show that the approach is transferable to other cancer driver genes, suggesting that the approach can be broadly utilized. Hence, this integrated platform not only provides insights into the biology of *TP53* mutations but also establishes a framework for studying the functional consequences of driver mutations and their therapeutic implications.

## Results

### Correction of TP53-R273H leads to growth disadvantage in multiple cancer cell lines

The *TP53*-R273H mutation is one of the most common hotspot mutations in cancer, found across various tumor types and usually co-occurring with a plethora of other (driver) mutations [41–44]. While it is found so frequently, it remains unclear whether correcting this mutation would lead to a similar depletion of mutant cells across different cancer types or if the response would vary due to the distinct tissue origins and genetic backgrounds of the tumors. To investigate this, we analyzed four cancer cell lines derived from a pancreatic ductal adenocarcinoma (PANC-1), epidermoid carcinoma (A431), colorectal adenocarcinoma (HT-29), and non-small cell lung adenocarcinoma (NCI-H1975), each harboring the *TP53*-R273H mutation together with numerous unique co-occurring mutations (Additional file 1: Table S1).

**Fig. 1:**
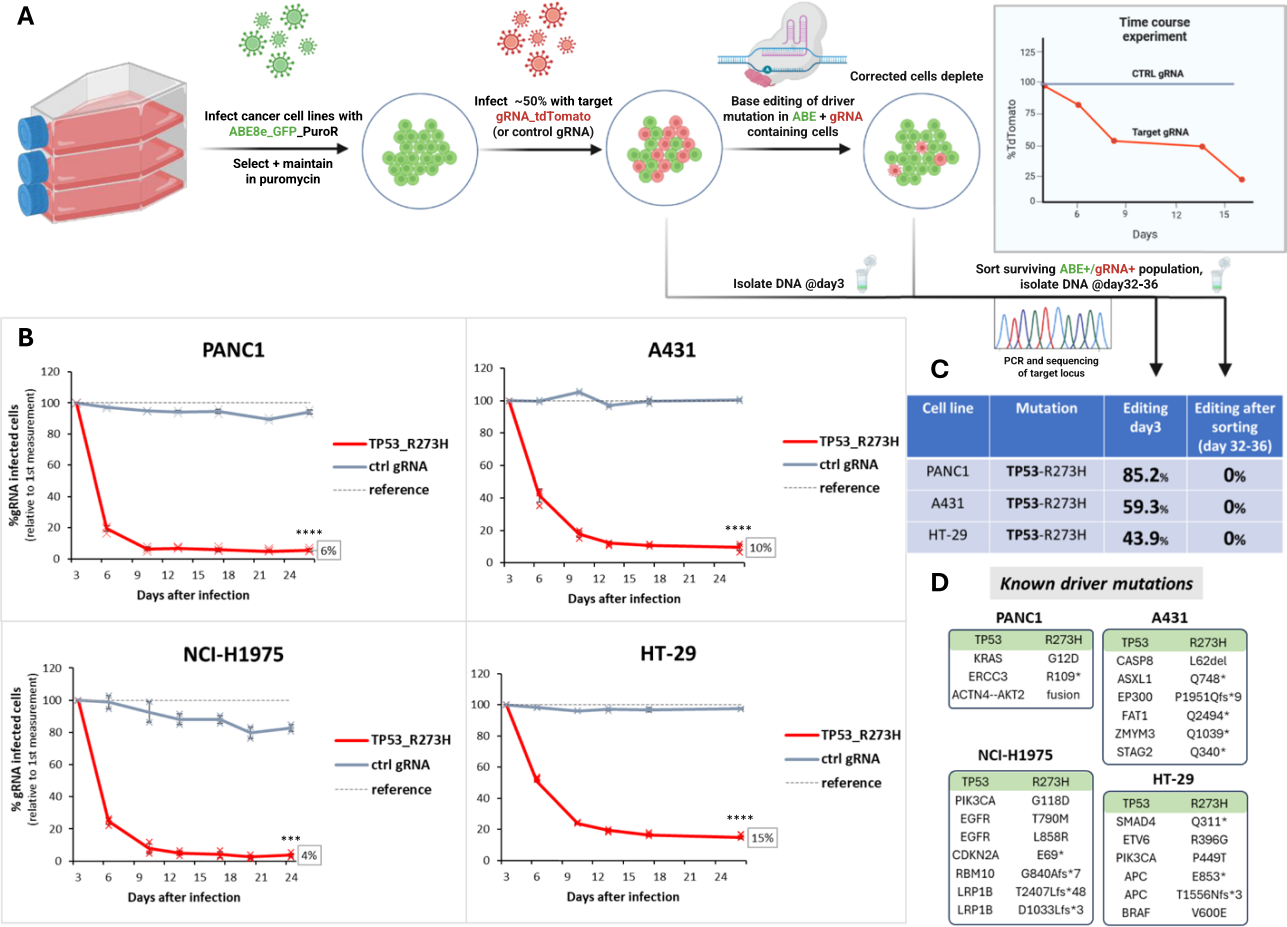
Correction of TP53-R273H in four different cancer cell lines. **A** Schematic overview of the experimental setup. Important steps are indicated by arrows. On day 3 post infection, the time course was started, and the ratio of tdTomato positive (= gRNA-expressing) versus tdTomato negative cells was measured every 3–6 days. The tdTomato percentages measured at day 3 were set to 100%. **B** Time course of the four indicated cell lines PANC-1, A431, HT-29, and NCI-H1975 for the *TP53*-R273H gRNA transduced cells (red) versus control gRNA transduced cells (gray). Reference = tdTomato level at day 3. Error bars represent mean + SD from independent infections in triplicates. *** indicates *p* < 0.001, **** indicates *p* < 0.0001. **C** Editing efficiency at the target loci of the corrected driver mutations. For day 3, DNA was taken from the mixed population (e.g., 50% infected with gRNA virus), and editing was normalized to the gRNA-expression level. For day 32–36, DNA was isolated from the sorted, tdTomato/GFP double-positive population. **D** High confidence driver mutations of the four lines, annotated as “oncogenic” or “likely oncogenic” in OncoKB [46, 47]

The four cell lines were infected with a lentivirus containing an adenine base editor (NG-ABE8e) [45] coupled to a GFP cassette, as well as a puromycin resistance gene. Following puromycin selection, the ABE-expressing cells were infected at a level of ~ 30–80% with another lentivirus containing both the gRNA for *TP53*-R273H correction as well as a second fluorescent protein (e.g., tdTomato). Hence, two competing populations, one expressing only the ABE (green) and another expressing both ABE and the R273H-correcting gRNA (green + red) were co-cultured in the same well. As a control, a gRNA targeting a functionally irrelevant adenine, as well as a non-targeting gRNA, were used in separate experiments to exclude possible effects due to infection with a lentivirus. The levels of green and green + red cells were then followed over time and red fluorescence was measured every 3–6 days for ~ 25 days (Fig. 1A).

All four cancer cell lines infected with the R273H gRNA-containing lentivirus exhibited a rapid decline compared to those infected with the control gRNAs (Fig. 1B, Additional file 2: Fig. S1), suggesting that correcting the mutation back to the wild-type sequence extensively impaired cell growth across all four lines. A rapid reduction in the *TP53*-R273H gRNA-expressing cells was observed at early time points, continuing until approximately day 10, after which the decline gradually plateaued. To confirm editing, we performed Sanger sequencing of a PCR fragment obtained from genomic DNA isolated from three of the lines 3 days post infection with the gRNA virus. *TP53*-R273H editing rates were measured between 44 and 85% (Fig. 1C), demonstrating the rapid and efficient correction of the driver mutation in these cells.

To determine co-occurring cancer driver mutations in the four cell lines, we extracted their mutational profile from the DepMap portal [48], the COSMIC Cancer Gene Census [49], and the OncoKB database [46, 47]. This analysis revealed a set of 132–365 overall mutational burden and 4–8 high confidence driver mutations per line, all of which were unique except for *TP53*-R273H (Fig. 1D, Additional file 1: Table S1). Hence, despite differences in tissue of origin and mutational profile, all four cell lines exhibited a uniform dependency on the R273H driver mutation.

After 10–15 days in culture, all of the corrected cell lines reached a plateau, where no further depletion was observed, despite the fact that the cells continued to express GFP and tdTomato (Fig. 1B). This was surprising, because presumably they should still express the ABE and the mutation-targeting gRNA. The inability to reach complete depletion could indicate that clones had emerged that can grow in the presence of wild-type p53, or that bystander edits had inactivated p53 expression altogether. To investigate these possibilities, we first sorted the surviving GFP +/tdTomato + cells, followed by gDNA extraction. Sanger sequencing of PCR fragments amplifying the hotspot mutation was then repeated. Intriguingly, no on-target or bystander editing could be observed in any of the samples (Fig. 1C), implying that there must be another reason for the ability of the cells to survive.

To investigate why the surviving double-positive cells remained unedited, we re-challenged one of the GFP +/tdTomato + sorted cell lines (PANC-1) by transfecting it with either ABE mRNA or synthetic *TP53*-R273H gRNA (Additional file 2: Fig. S2A). Transfection with the ABE mRNA did not lead to any editing. In sharp contrast, cells transfected with the R273H gRNA and the control gRNA showed more than 70% A-to-G conversion (Additional file 2: Fig. S2B + D). Moreover, in comparison to the ABE mRNA and control gRNA transfected cells, we observed a clear depletion of the cells transfected with the driver mutation-correcting gRNA (Additional file 2: Fig. S2C), demonstrating that these cells are still sensitive to *TP53* correction. This result indicates that in the surviving cells, the base editor is functionally intact, whereas the gRNA does not seem to be expressed properly. Indeed, we observed a greater than 50,000-fold reduction in *TP53* gRNA expression in sorted GFP+/tdTomato+ cells compared to freshly *TP53* gRNA-infected cells, suggesting that silencing of the U6 promoter may underlie the lack of editing (Additional file 2: Fig. S3).

We conclude that correction of the *TP53*-R273H mutation induces a strong and rapid depletion of corrected cells. This effect is comparable across different cancer cell lines and independent of their mutational profile. Because we observed no editing in the surviving cells, this might indicate that correction of the *TP53*-R273H mutation could be an event that is hard to overcome for the cells and to acquire resistance against. Taken together, these results support the notion of the same p53 mutation being critical across different tumor types, highlighting the role of p53 as a central tumor suppressor.

### Correction of TP53-R273H restores conserved tumor-suppressive transcriptional programs

**Fig. 2:**
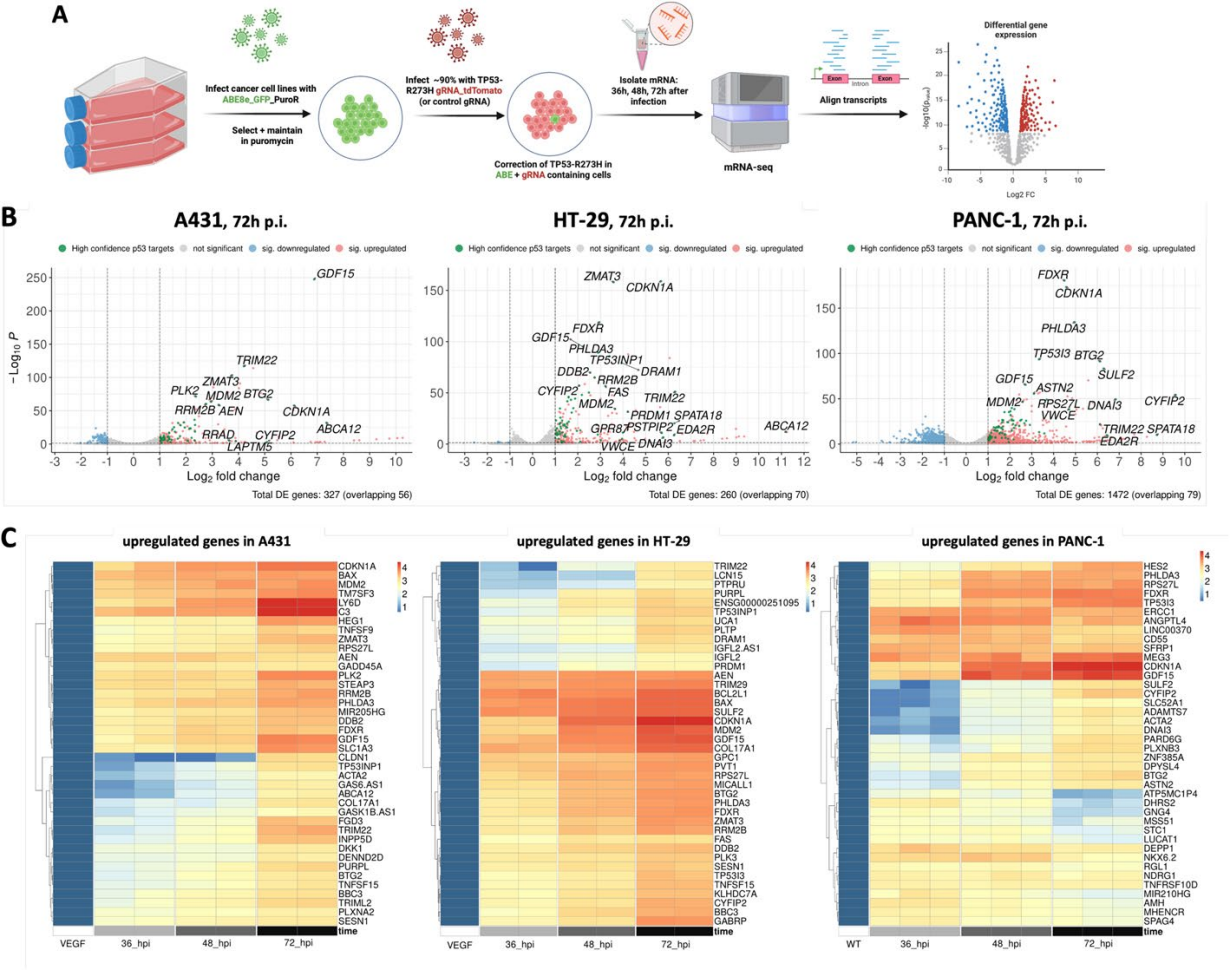
RNA-Seq reveals differentially expressed genes in *TP53*-R273H corrected cells. **A** Schematic workflow with important steps indicated by arrows. **B** Volcano plots of DE genes 72 h post infection (*p* < 0.05, fc > 2). Gene names overlapping with a census of 116 core p53 targets from Fischer (2017) [50] are indicated. **C** Heatmaps of upregulated genes by cell line. *X*-axis: independent replicates used for mRNA-seq, sorted by time point (36–72 h p.i.). Genes were grouped using hierarchical clustering

To start unraveling the molecular mechanisms underlying the detrimental effects of *TP53*-R273H correction across different cell lines, we performed RNA-seq experiments on three R273H-mutant lines (PANC-1, HT-29, A431). Cell lines expressing the ABE were infected with a virus expressing either a control gRNA or one targeting the driver mutation, and RNA was isolated at 36, 48, and 72 h post infection (p.i.). Transcriptome analysis via RNA-seq was then performed to identify differentially expressed (DE) transcripts (Fig. 2A).

To first evaluate the efficiencies of *TP53* correction, we quantified high-quality reads from transcripts carrying the wildtype versus mutant sequence. Consistent with the previous experiments (Fig. 1C), we detected editing rates ranging from 21 to 35% at 36 h, increasing to 47–90% at 72 h post infection, confirming efficient base editing across all RNA samples (Additional file 2: Fig. S4).

Next, we determined DE genes after *TP53* correction at the different time points. At the earlier ones (36 h and 48 h p.i.), comparatively few transcripts were significantly changed in their expression in all three cell lines, consistent with the time required to complete the editing of the DNA and subsequent transcriptional changes to occur (Additional file 2: Fig. S5A). Changes in the transcriptional profiles substantially increased at 72 h p.i., when ~ 250–1500 genes were found to be differentially expressed in the three corrected cell lines (Fig. 2B). The majority of DE genes were upregulated, indicating that re-expression of the wild type p53 protein predominantly restored its main role as a transcriptional activator [51] (Fig. 2B). Notably, we observed a substantial overlap in DE genes across the different cell lines. For instance, 188 (72%) and 160 (62%) of the 260 DE genes in HT-29 were shared with those in PANC-1 and A431, respectively (Additional file 2: Fig. S5B). Although differences in splicing, transcript abundance, or genome organization across the cell lines cannot be excluded, this finding suggests that correcting the *TP53* mutation induces remarkably similar transcriptional changes, despite the heterogeneity in co-occurring mutations and the distinct gene expression programs operating in these cell lines (Additional file 2: Fig. S5C).

To gain deeper insight into the transcriptional changes observed, we first examined the genes that were most significantly upregulated across the three cell lines (Fig. 2B). As expected, this included several key regulators of p53-mediated response, consistently identified in all three lines. Prominent among these was *CDKN1A*, an early and well-characterized p53 target with crucial roles in cell cycle regulation and cellular senescence [52, 53]. Additionally, transcripts associated with cell cycle arrest [54], such as *BTG2*, *ZMAT3*, *GDF15*, and *PLK2/3* [55–59], were notably upregulated (Fig. 2B). Apoptosis-promoting genes [54], including *BBC3* (*PUMA*), *BAX*, *PHLDA3*, *CYFIP2*, and *TP53I3* [60–64], were also prominently induced, underscoring the activation of multiple p53-dependent pathways (Fig. 2B). Moreover, investigating the different time points allowed us to distinguish early and late responders within the upregulated genes (Fig. 2C, Additional file 2: Fig. S5A) [54].

We then examined the most significant downregulated transcripts. Among the downregulated genes, *ESCO2*—involved in S-phase progression [65]—was the only transcript consistently suppressed across all three cell lines. However, a greater number of significantly downregulated genes were identified in at least two out of three lines, many of which are integral to cell cycle regulation and mitosis (Fig. 2B). This finding aligns with *TP53*’s function in enforcing cell cycle arrest and maintaining genomic stability by suppressing proliferative and mitotic pathways [66].

#### Comparison to meta-analyses uncovers high confidence p53 targets

Over the years, numerous individual studies and high-throughput analyses have been conducted to classify p53 target genes. However, these efforts have demonstrated limited consistency. For example, a comparative analysis of 16 datasets from 13 genome-wide studies of p53 target genes revealed that only two genes were consistently identified across all datasets [50]. This lack of overlap suggests a high prevalence of both false positives and false negatives within these datasets. To address this issue and establish a more reliable set of p53 target genes, a meta-analysis integrating both individual studies and high-throughput datasets has been performed. This analysis identified 116 high-confidence p53 target genes [50].

**Fig. 3:**
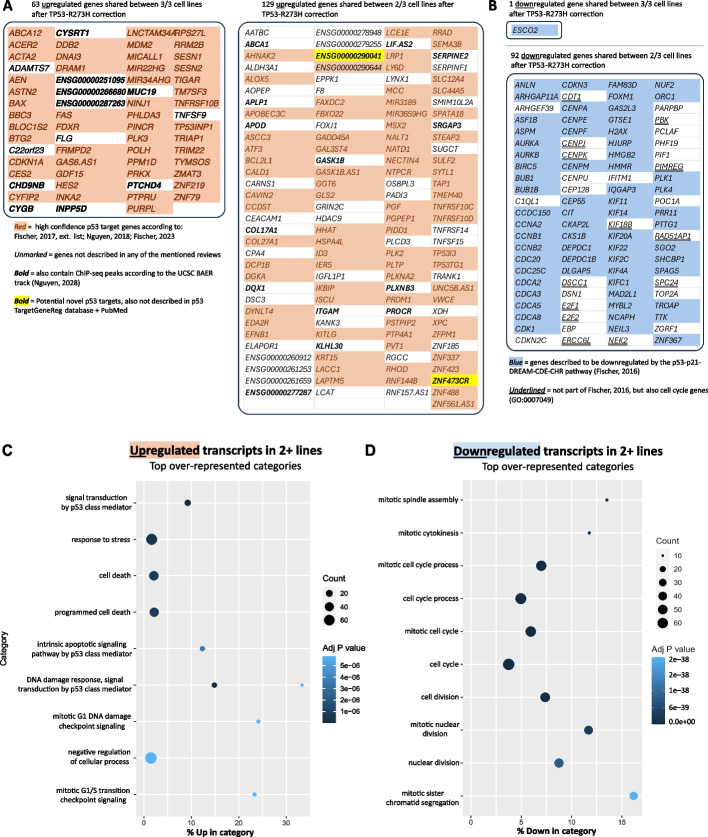
Analysis of differentially expressed genes at 72 h after *TP53*-R273H correction in A431, HT-29, and PANC-1 cells. **A** Consensus of upregulated genes in all three cell lines (left) or in two of the three lines (right). High confidence p53 target genes are highlighted in light red if they overlap with either [63, 67] or [68]. Genes that overlap with ChIP-seq peaks from the UCSC p53 BAER track [67] are shown in bold. **B** Downregulated genes in all three lines (top) or in two of the three lines (bottom). Genes described to be downregulated by the p53-p21-DREAM-CDE-CHR pathway in [66] are marked in blue. Genes described to have a role in cell cycle regulation are underlined. See also: Additional file 3: Table S2. **C** + **D** Gene ontology enrichment analysis of genes up-/downregulated in at least two of the three lines

To compare our dataset with this established census of high confidence p53 target genes, we first looked at the individual cancer lines and detected striking similarities: In A431, a total of 56 of the 116 genes described to be the core transcriptional program were found to be upregulated. In HT-29 and PANC-1, this number was even higher with 70 and 79 genes overlapping (Additional file 2: Fig. S6, Additional file 3: Table S2). Next, we compiled lists of commonly upregulated and downregulated genes from our transcriptional profiles. A total of 192 shared genes were upregulated in at least two of the three cancer cell lines, with 63 genes consistently induced across all three lines (Fig. 3A). Gene ontology enrichment analysis of these upregulated transcripts showed a significant enrichment of pathways that are associated with p53 function (Fig. 3C). These findings suggest that correcting *TP53* hotspot mutations, followed by transcriptome profiling, represents an effective strategy for uncovering p53 target genes.

Fewer consensus transcripts were observed to be downregulated, aligning with p53’s primary function as a transcriptional activator, where downregulation typically occurs indirectly via the p53-p21-DREAM pathway [51, 66]. A total of 92 genes were downregulated in at least two of the three lines, most of which play critical roles in cell cycle regulation and mitosis (Fig. 3B). This included key regulators of G2/M progression such as AURKA, AURKB, and PLK1 [69]. Gene ontology enrichment analysis of these (92) downregulated transcripts confirmed a significant enrichment of “cell cycle” and “mitosis”-related terms (Fig. 3D), consistent with prior findings that most downregulated genes are indirect targets of the p53-p21-DREAM-CDE-CHR axis [66]. Interestingly, our list of downregulated genes includes additional genes implicated in cell cycle regulation such as CDKN2C, CDT1, CENPJ, DSCC1, DSN1, E2F1 and E2F2, ERCC6L, KIF18B, NEK2, PBK, PIMREG, RAD51AP1, SPC24, and ZGRF1, suggesting that these genes might also be targeted for downregulation by the p53-p21-DREAM pathway.

To identify potential novel p53 transcriptional targets, we compared our dataset to three comprehensive p53 target gene lists: (1) an extended list of targets identified in at least three independent studies [50]; (2) a meta-analysis of 41 genome-wide p53 ChIP-seq datasets, defining a p53 core cistrome [67]; and (3) an analysis focusing on lncRNAs as p53 targets [68]. After filtering out genes already identified in these datasets, we identified 60 upregulated genes that had not been previously described as p53 targets, 13 of which were consistently upregulated in all three cell lines (Fig. 3A, Additional file 3: Table S2). Recognizing the challenge of distinguishing direct p53 targets from secondary transcriptional effects, we further refined this list by examining publicly available p53 ChIP-seq data using the p53 BAER track [67]. This analysis revealed 25 upregulated genes with ChIP-seq peaks detected in at least two independent datasets, suggesting direct binding by p53. Of these, nine genes were upregulated in all three cell lines (Fig. 3A). While some of these genes have been reported in isolated studies, they have not yet been incorporated into high-confidence p53 target gene censuses. Our data suggests that these 25 transcripts warrant reconsideration as bona fide p53 targets. Among the most compelling candidates are PTCHD4, CYSRT1, and INPP5D, all of which were upregulated in all three cell lines. Additionally, ENSG00000251095 (LOC124900602) and ENSG00000287263 (ANXA2R-OT1) were identified, both containing perfect 20-mer p53 response elements within their respective ChIP-seq peaks (Additional file 2: Fig. S7A).

Interestingly, we also identified a significant number of long non-coding RNAs (lncRNAs) among the upregulated transcripts, underscoring the growing recognition of lncRNAs as integral components of p53 signaling pathways [68, 70, 71]. For example, PINCR [72], PURPL [73], TYMSOS [74], MIR22HG [75], and MIR34HG [76] were consistently upregulated, reflecting the broader transcriptional role of *TP53* beyond protein-coding genes. In this regard, we also discovered two novel lncRNAs, ZNF473CR and ENSG00000290041, with measured p53 ChIP-peaks (Additional file 2: Fig. S7B) that were upregulated in two of the three cell lines following *TP53* correction (Fig. 3A). To the best of our knowledge, these lncRNAs have not been previously described as p53 targets, highlighting new avenues for exploring the role of non-coding RNAs in p53-mediated transcriptional regulation.

### Correction of TP53-R175H similarly restores p53 tumor-suppressive programs

**Fig. 4:**
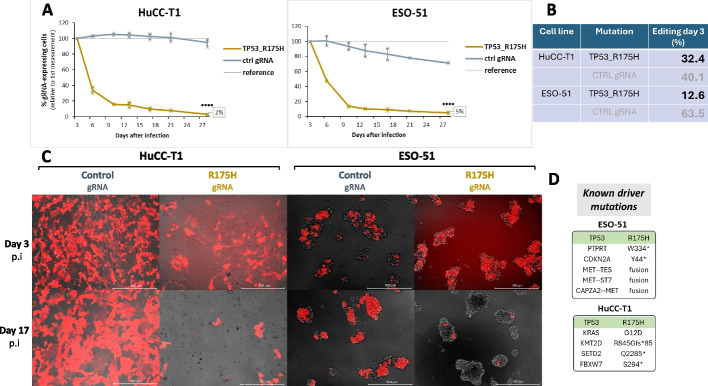
Correction of *TP53*-R175H in two different cancer cell lines. **A** Time course of HuCC-T1 and ESO-51 cells for the *TP53*-R175H gRNA transduced cells (brown) versus control gRNA transduced cells (gray). Reference = tdTomato level at day 3. Error bars represent mean + SD from independent infections in triplicates. **B** Editing efficiency at day 3 after infection with *TP53*-R175H-repairing gRNA lentivirus. DNA was taken from the mixed population (e.g., 50% infected with gRNA virus), and editing was normalized to the gRNA-expression level. **C** Representative fluorescent images of control or R175H gRNA-expressing cells at day 3 and day 17 after infection. Note the strong depletion of red cells at day 17 for the R175H gRNA transduced cells. **D** High confidence driver mutations of the two lines, annotated as “oncogenic” or “likely oncogenic” in OncoKB [46, 47]

Motivated by the results showing that correction of the *TP53*-R273H hotspot mutation leads to strong depletion in corrected cells, we sought to investigate if the same applies to R175H, a second *TP53* hotspot mutation that has been shown to represent a “conformational” mutation [77]. Thus, two cancer cell lines originating from different tissues and containing the R175H mutation were corrected in the equivalent way: HuCC-T1 (cholangiocellular carcinoma) and ESO-51 (esophageal adenocarcinoma) (Fig. 4).

Similar to the effects observed with *TP53*-R273H correction, the correction of R175H also had a strong and rapid detrimental impact on cells expressing both ABE and the gRNA targeting the driver mutation, with cells declining to a plateau around days 10–15 post-infection (Fig. 4A, Additional file 2: Fig. S1). After 4 weeks in culture, only ~ 3–5% of GFP/tdTomato-expressing cells remained, indicating a high sensitivity to the correction of this driver mutation in both cell lines. Sanger sequencing of the target locus 3 days post infection revealed 13–32% A-to-G conversion, confirming editing of the target base (Fig. 4B). When comparing the mutational profile, we again found no similarities between the lines except for the R175H hotspot mutation (Fig. 4D, Additional file 1: Table S1). Notably, despite these differences in tissue origin and co-mutation profiles, both cell lines exhibited comparable sensitivity to *TP53*-R175H correction. This result supports the notion that *TP53* hotspot mutations may maintain oncogenesis in a manner that is independent of cell type and co-occurring mutations.

**Fig. 5:**
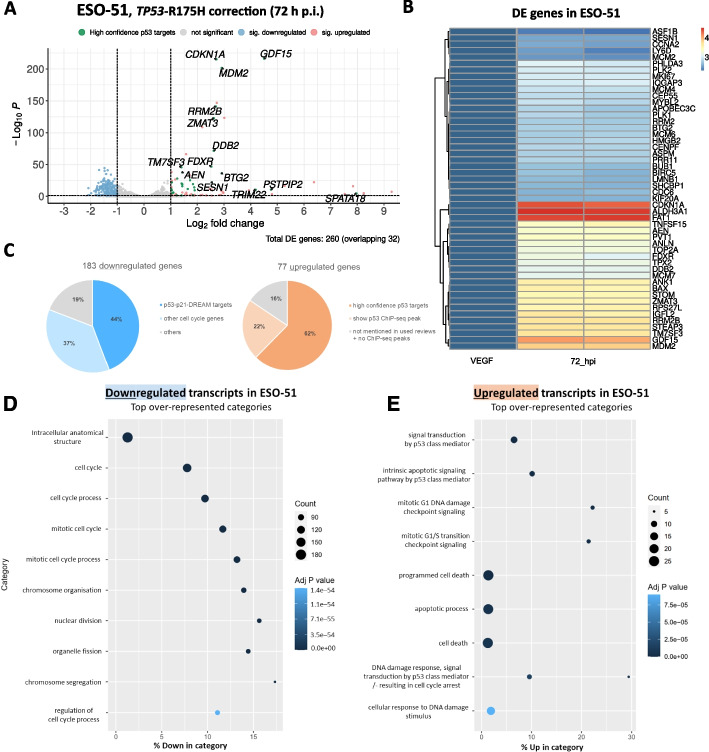
Analysis of differentially expressed genes at 72 h after *TP53*-R175H correction in ESO-51 cells. **A** Volcano plot of DE genes 72 h post infection (*p* < 0.05, fc > 2). Gene names overlapping with a census of 116 core p53 targets from Fischer (2017) [50] are indicated. **B** Heatmap of differentially expressed (DE) genes. *X*-axis: independent replicates used for mRNA-seq. Genes were grouped using hierarchical clustering. **C** Left: analysis of upregulated genes. High-confidence p53 target genes are highlighted in red if they overlap with either of [50, 64, 66]. Genes that overlap with ChIP-seq peaks from the UCSC p53 BAER track [67] are highlighted in light red. Right: Analysis of downregulated genes. Genes described to be downregulated by the p53-p21-DREAM-CDE-CHR pathway in [66] are marked in blue. From the remaining genes, those that are part of the “cell cycle” term GO:0007049 are marked in light blue. (D + E) Gene ontology enrichment analysis of up-/downregulated genes

To investigate whether the conserved p53 tumor-suppressive program we observed after correction of the R273H mutation also extends to correction of the R175H mutation, we performed mRNA-seq for one of the two lines containing the R175H mutation (ESO-51, at 72 h p.i.). Correction of the R175H mutation was confirmed on transcript level (58%) (Additional file 2: Fig. S8), resulting in 260 DE genes passing the significance and fold change thresholds (Fig. 5A + B). The similarities in p53 response were stunning: Among the upregulated genes, 87% were also upregulated in at least one of the R273H corrected lines (Additional file 4: Table S3), with 63% of the upregulated genes described as high confidence p53 targets [50, 67, 68] (Fig. 5C). Looking at the downregulated genes, we observed a similar pattern: an astonishing 93% were also downregulated in at least one of the R273H-corrected lines. Moreover, clear activation of the DREAM pathway was observed, as well as downregulation of a plethora of other cell cycle genes. Gene ontology analysis confirmed the enrichment of terms associated with p53 function (Fig. 5D + E). To identify potential mutation-specific DE genes, we sought to compare DE genes following R273H vs R175H repair. Interestingly, a total of twenty-six transcripts were upregulated in all the lines following the R273H mutation correction but were not differentially expressed in ESO-51 (Additional file 4: Table S3). These transcripts might indicate a mutation-specific signature. However, at this point we cannot exclude other reasons for this observation, such as clonal variation or differences in cellular context. Performing R175H correction in additional cancer cell lines, followed by RNA-Seq experiments could help determine whether the observed transcriptional changes are indeed mutation-specific. Altogether, correction of the R175H mutation resulted in a markedly similar response to the R273H correction, with canonical p53 response being reinstated as well as up-/downregulated genes largely overlapping with the R273H lines.

### Validation of the platform through correction of SMAD4-Q311*, PTEN-R233*, and KRAS-G12D 

**Fig. 6:**
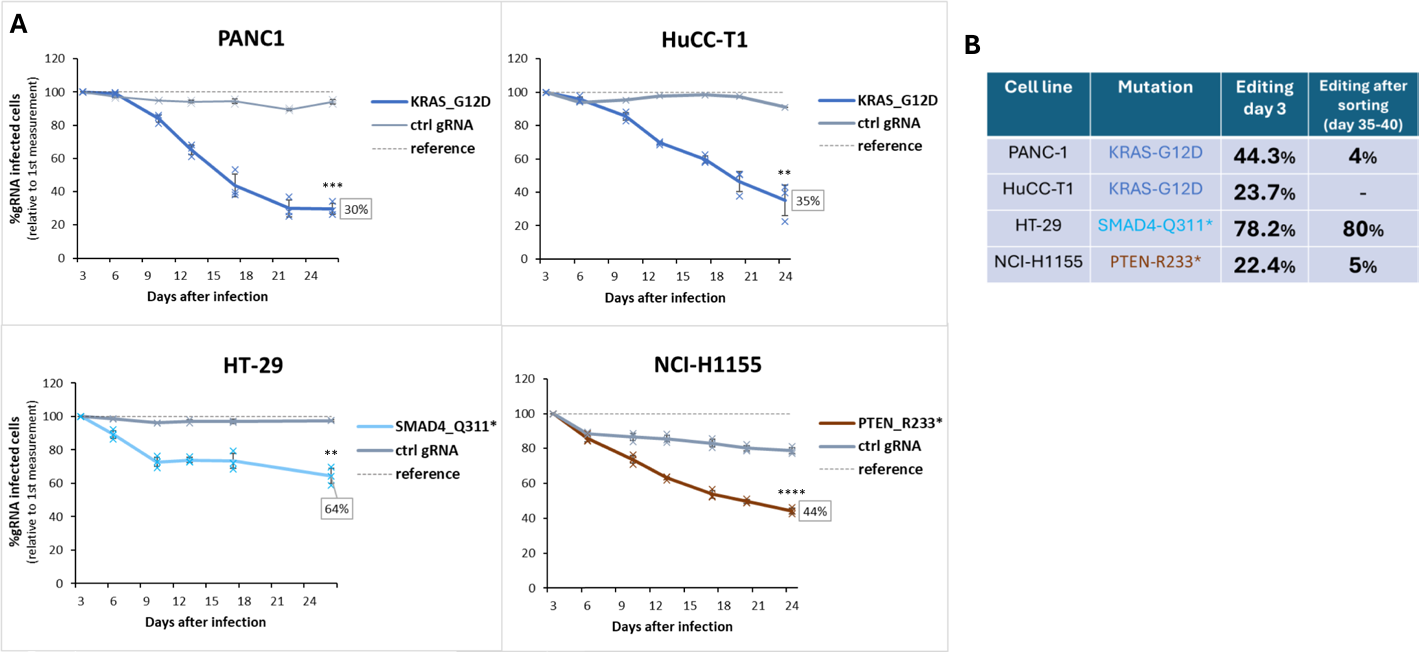
Correction of miscellaneous driver mutations affects cell growth in different ways. **A** Time course of indicated cell lines and indicated mutation correcting gRNAs versus control gRNA transduced cells (gray). The ratio of tdTomato (= gRNA) positive versus tdTomato negative cells was measured starting at day 3 post infection, every 3 to 6 days. The tdTomato percentages measured at day 3 were set to 100%. Reference = tdTomato level at day 3. Error bars represent mean + SD from independent infections in triplicates. ** indicates *p* < 0.01, *** indicates *p* < 0.001, **** indicates *p* < 0.0001. **B** Editing efficiency at the target loci of the corrected driver mutations. For day 3, total DNA was taken from the mixed population and normalized to the gRNA-expression level. For day 35 to 40, DNA was isolated from the sorted, tdTomato/GFP double-positive population

Having shown that correction of driver mutations can be used to study hotspot mutations of p53, we wanted to extend our approach towards other commonly mutated cancer driver genes. For this purpose, we investigated correction of *KRAS*-G12D in PANC-1 and HuCC-T1 cells, *SMAD4*-Q311* in HT-29, as well as *PTEN*-R233* in the NCI-H1155 line. A time course was run in the same manner as described before, with the cancer lines being infected first with an ABE-containing virus (green), followed by subsequent infection with a gRNA-containing second virus (red). The level of gRNA-expressing population was then followed again over time (Fig. 6A):

Depletion dynamics differed markedly from correction of the *TP53*-R273H and R175H mutations. In contrast to the *TP53* experiments, correction of other driver mutations did not induce comparably strong effects. For instance, correction of *KRAS*-G12D led to a reduction of gRNA-expressing cells to ~ 30–35%, which occurred with a slower, more linear progression in both PANC-1 and HuCC-T1 cell lines. Similarly, correction of *SMAD4*-Q311* and *PTEN*-R233* resulted in more moderate depletions, with 64% and 44% of gRNA-containing cells surviving, respectively (Fig. 6A, Additional file 2: Fig. S1). This weaker depletion is unlikely due to differences in editing efficiency, because equally strong or even stronger A-to-G conversion compared to the *TP53* corrections was detected for these drivers, ranging from 22 to 78% (Fig. 6B). In contrast, KRAS and PTEN each regulate distinct, individual growth-controlling pathways (RAS–RAF–MEK–ERK for KRAS, PI3K–AKT for PTEN), which may explain their comparatively weaker depletion phenotypes. Nonetheless, this interpretation remains speculative, and further investigation is required to delineate the relative contribution of pathway-specific effects versus broader tumor suppressor network activation.

Intriguingly, sorting of the surviving ABE/gRNA-expressing populations at the end of the time course revealed residual editing across varying levels (4–80%, Fig. 6B), suggesting that corrected cells can survive much longer upon re-expression of the wild type allele, or that resistant clones have emerged in the population. Particularly striking was the proportion of corrected cells remaining in the *SMAD4* edited cells (80%). Closer inspection of the curve progression revealed that after an initial drop of *SMAD4* correcting gRNA expressing cells up to day 9, no further depletion was observed at later time points (Fig. 6A). Together with the high proportion of *SMAD4* corrected cells at the end of the experiment, this result suggests that after an initial depletion, the cells adapted to re-expression of restored *SMAD4* and continued proliferating without any growth disadvantage.

**Fig. 7:**
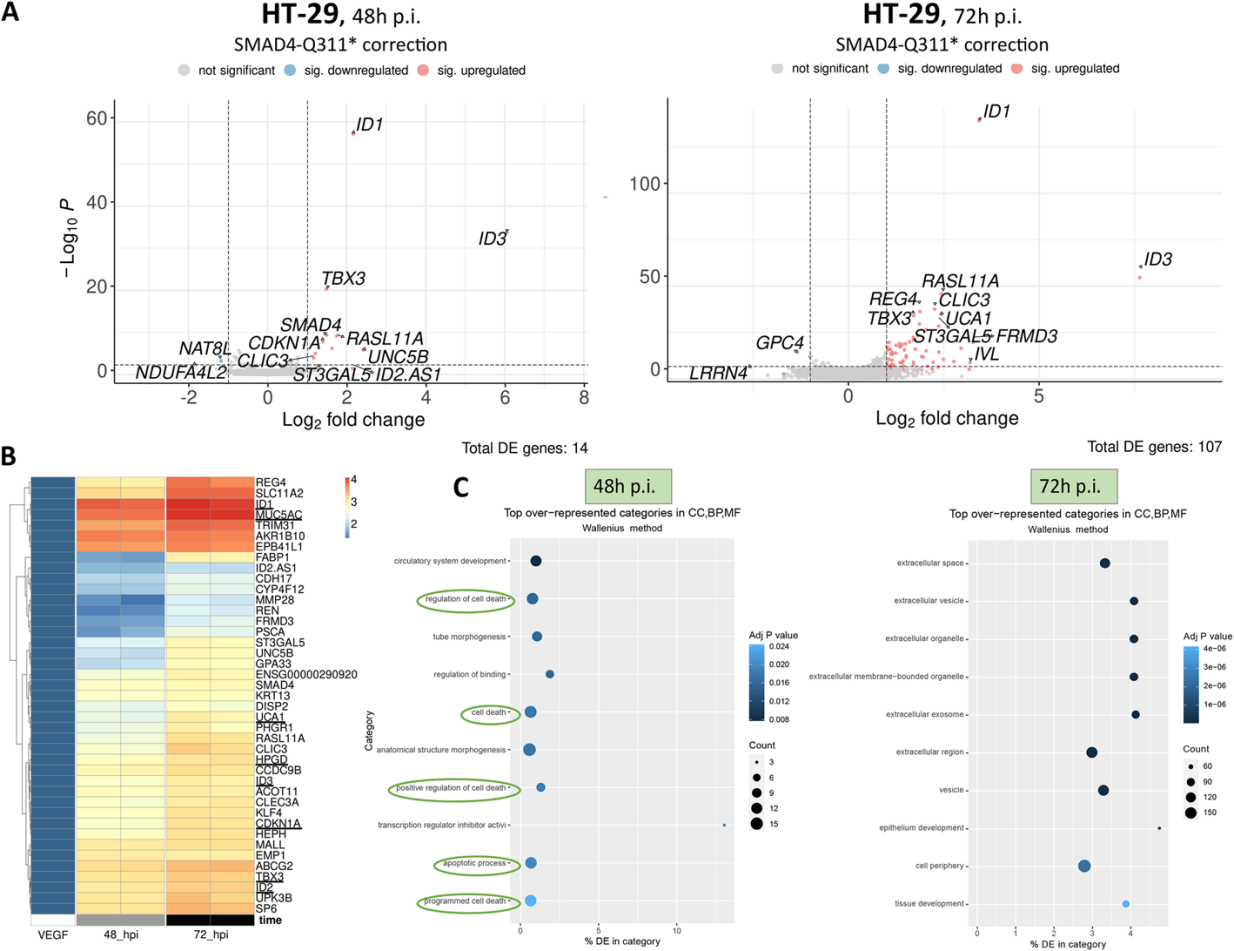
Differentially expressed genes after correction of *SMAD4*-Q311*. ABE-expressing cells were infected with *SMAD4*-Q311* repairing gRNA lentivirus. Total RNA was isolated 48 h and 72 h after infection and analyzed by RNA-seq. **A** Volcano plots of DE genes 48h and 72 h post infection (*p* < 0.05, fc > 2). Top 12 significant genes are highlighted. **B** Heatmap of upregulated genes by cell line, all time points merged, grouped using hierarchical clustering. *X*-axis: Independent replicates used for RNA-seq, sorted by timepoint (48 h + 72 h p.i.). Representative targets of TGF-beta pathway underlined. **C** Gene ontology enrichment analysis of DE genes at indicated time points

In order to find a possible explanation for this phenotype, we decided to analyze the transcriptome of *SMAD4*-Q311* corrected cells at 48 h and 72 h post infection via RNA-seq as described before. At these time points, we observed 14 and 107 DE genes, respectively (mostly upregulated). Inspecting the transcripts for editing on RNA level, we detected an astonishing 91 to 98% correction of the target adenine at 48 h and 72 h p.i., respectively. This high rate of editing at the RNA level is likely driven by nonsense-mediated decay of the mutated *SMAD4* transcript, which in turn leads to a higher stability of the corrected transcripts, ultimately resulting in the observed editing rate approaching 100%. This hypothesis is supported by the analysis of the RNA-seq data at the earlier time point, which revealed *SMAD4* as one of the most upregulated transcripts (Fig. 7A).

Gene ontology enrichment analysis at the early time point revealed a strong enrichment of categories related to apoptosis and cell death, providing a possible explanation for the depletion initially seen (Fig. 7C). Interestingly, in the gene ontology enrichment analysis for the later time point, these categories had vanished and were now dominated by extracellular and developmental pathways, possibly reflecting that the cells had adapted to the correction and re-expression of *SMAD4*. Markedly, members of the ID protein family (ID1, ID2, and ID3) were strongly upregulated after correction (Fig. 7A + B), consistent with their role as *SMAD4* target genes [78, 79] in the TGF-beta pathway [80]. In fact, other TGF-beta components, such as UCA1 [81, 82], HPGD [83], MUC5AC [84], and TBX3 [85] were also upregulated (Additional file 3: Table S2). Future work could investigate if the upregulation of these factors plays a role in the adaptation process [86, 87].

Altogether, by utilizing base editing, we corrected a variety of known cancer driver mutations and measured subsequent depletion over time. This enabled us not only to uncover dependencies of a given cancer on a common driver mutation but also provided a qualitative comparison between different drivers within the same cancer line. Given the critical importance of uncovering the potential vulnerabilities of cancers on their respective driver mutations, e.g., in clinical diagnostics, our system provides a valuable tool for making meaningful comparisons.

## Discussion

Base editing has emerged as a transformative technology, enabling precise and efficient nucleotide modifications without inducing double-stranded breaks [88]. This approach has significantly advanced various scientific fields, including genetic disease modeling [89], functional genomics [90–92], and potential therapeutic interventions [93–95]. While initial studies have begun to explore the use of base editing for investigating cancer driver mutations [37, 39, 40] the full potential of this technology for systematically characterizing their functional and transcriptional consequences remains to be fully realized.

In this study, we utilized the power of adenine base editing to functionally and transcriptionally profile cancer driver mutations, with a primary focus on *TP53* hotspot mutations. By correcting *TP53*-R273H and *TP53*-R175H mutations in cancer cell lines derived from diverse tissues, we were able to uncover both the phenotypic dependencies on mutant *TP53* expression and the restoration of conserved tumor-suppressive transcriptional programs upon correction. This combined approach not only illuminated the critical role of *TP53* mutations in sustaining oncogenic phenotypes but also revealed the robustness of the reinstated p53-dependent regulatory networks. Recently, small-molecule inhibitors have been employed to investigate p53-dependent transcriptional responses [96, 97]. Comparing the transcriptional programs elicited by genetic correction via base editing with those induced by pharmacological activation could provide deeper insights into the context-specific dynamics and therapeutic potential of p53 pathway reactivation.

Considering the extensive efforts by numerous research groups to assemble comprehensive lists of p53 target genes [50, 54, 67, 68], we believe that the relatively simple platform described in this manuscript offers a straightforward and scalable approach for identifying transcriptional targets of additional cancer driver genes. In this context, comparing the expression profiles of corrected cell lines harboring different p53 hotspot mutations, such as R273 and R175H, could offer valuable insights.

Notably, we predict that the versatility of this platform extends beyond transcriptomics, as it can be readily adapted to integrate other omics technologies, including proteomics [98] and metabolomics [99]. Correcting the same cancer driver mutation in different cell lines, followed by proteomic and metabolomic profiling, could uncover downstream effects at the protein and metabolic levels that are not captured by transcriptomic analysis alone. This multi-omics approach would have the potential to reveal novel signaling pathways, post-translational modifications, and metabolic reprogramming events associated with specific mutations, providing a more comprehensive understanding of the functional consequences of cancer driver alterations. Ultimately, such integrative analyses could identify new biomarkers and therapeutic vulnerabilities, further advancing precision oncology.

In the current study, we focused on the application of adenine base editors (ABEs) due to their demonstrated high efficiency and minimal propensity for off-target editing [45, 95, 100, 101]. ABEs are particularly well-suited for correcting cancer-associated mutations, as a significant proportion of driver mutations involve A·T to G·C transitions. In fact, nearly half of all known cancer driver mutations have been described to be potentially addressable using ABEs [36, 102]. For instance, when looking at *TP53*, five of the seven most common hotspot mutations are addressable using ABEs (Additional file 2: Fig. S9B). Nevertheless, the system has several limitations, including bystander editing, which is particularly pronounced when employing the ABE8e system. Given the broad editing window of ABE8e, which exhibits significant A-to-G conversion at gRNA positions 3–8 [45, 95, 100, 101], other adenine residues within the gRNA region are also likely to be edited. These additional edits may alter codons, potentially resulting in protein variants that lack wild-type function. To alleviate this problem, ABEs with a more narrow editing window, such as the ABE9 system [103], which almost exclusively targets positions 5–6, can be used. Furthermore, if low levels of detrimental bystander edits are unavoidable, employing single-cell RNA sequencing would allow untangling on-target from bystander editing in individual cells. Moreover, we anticipate that other base editing technologies, such as cytosine base editors (CBEs) [104, 105] and glycosylase-based base editors (GBEs) [106], could similarly be applied to expand the range of targetable mutations. CBEs enable precise C·G to T·A conversions, while GBEs facilitate T·A to G·C and C·G to G·C substitutions [107], offering complementary capabilities to address mutations not targetable by ABEs. In addition, for more complex mutations, including insertions, deletions, or transversions that are beyond the scope of current base editors, prime editors may represent a powerful alternative [108, 109].

Our platform’s versatility was validated through the correction of additional cancer-associated mutations in *SMAD4*, *PTEN*, and *KRAS*, demonstrating its broader applicability in dissecting the functional roles of diverse genetic alterations (Additional file 2: Fig. S9). Interestingly, we observed distinct progression dynamics following the correction of these mutations. While *TP53* correction resulted in a strong and rapid decline of corrected cells across multiple cancer cell lines, the correction of *SMAD4*, *PTEN*, and *KRAS* mutations exhibited more gradual declines, with less pronounced growth disadvantages that did not reach the same levels observed for *TP53*. Of note, at least one of the cell lines (A431) used in our study is *TP53* hemizygous [110], with the other five lines being either homozygous or hemizygous due to loss of the wildtype allele (variant allele frequency of 100% of the respective R273H or R175H mutations) [111–115], indicating that correction of a single allele is sufficient to induce prominent depletion of the cells. The observed differences suggest that the oncogenic dependencies and cellular consequences of driver mutations are highly driver-specific, reflecting the unique biological roles and downstream signaling pathways associated with each gene. The strong selective pressure against *TP53* correction likely reflects the central role of p53 in maintaining genomic integrity and suppressing tumorigenesis, whereas the more moderate effects observed for *SMAD4*, *PTEN*, and *KRAS* may be due to redundancy in signaling pathways or compensatory mechanisms within the cancer cells. More research is required to investigate this, but if confirmed, these findings hold significant potential for implementation in precision oncology. Intriguingly, incorporating mutation correction with single-cell RNA sequencing could offer not only deeper insights in gene network hierarchy and untangle confounding bystander edits, but potentially also reveal different p53 programs of distinct cell populations within the same cancer cell line.

Translating this base editing platform to patient-derived cancer organoid cultures [116, 117] could enable the functional assessment of correcting specific driver mutations in a patient-specific manner. Such an approach may offer valuable diagnostic and prognostic insights by distinguishing mutations essential for tumor maintenance from those with less impact. Furthermore, differential responses observed through this system could help predict patient-specific treatment outcomes, informing the development of tailored therapeutic strategies. Integrating these functional insights with genomic, transcriptomic, and multi-omic profiling could refine patient stratification based on tumor-specific mutational dependencies, ultimately guiding more precise and effective cancer treatments. Beyond diagnosis, the ultimate goal of oncology is to eradicate cancer cells, possibly through correction of cancer driver mutations in vivo. Notably, rapid advancements in the development of efficient in vivo delivery systems for genome editing tools [40, 118, 119] may further accelerate this transformation, bringing base editing closer to its potential as a therapeutic strategy for personalized oncology. Among emerging approaches, the delivery of base editor mRNAs in combination with chemically synthesized sgRNAs using lipid nanoparticles (LNPs) represents a particularly promising strategy. This method offers a transient yet efficient platform for genome editing that restricts exposure to the genome editing components, an important consideration for clinical translation. Indeed, recent studies have documented significant progress in applying LNP-based delivery for therapeutic genome editing [95, 120]. In parallel, engineered virus-like particles have also gained attention as non-integrating vectors capable of mediating in vivo delivery with high specificity and translational potential [121].

Finally, compared to conventional cancer treatments, which are often associated with significant toxicity and the risk of secondary malignancies, base editor-based therapies may offer a more targeted and well-tolerated alternative. Encouragingly, recent clinical trials involving base editing technologies have demonstrated favorable safety profiles [122]. As such, the future development of BE-based therapeutic approaches, potentially in combination with other interventions (e.g., immune therapy), warrants consideration in the context of precision oncology.

## Conclusions

Our study establishes a robust framework for investigating the functional and transcriptional impact of cancer hotspot mutations through adenine base editing. By correcting *TP53* hotspot mutations in diverse cancer cell lines, we demonstrated that mutant *TP53* expression is essential for sustaining oncogenic phenotypes, while its correction restores a highly conserved tumor-suppressive transcriptional program. Notably, these effects were consistent across different tissue types and independent of co-occurring mutations, underscoring the existence of a shared p53-dependent regulatory network. Beyond *TP53*, our platform’s applicability extends to other cancer-associated mutations, revealing gene-specific differences in oncogenic dependencies. Furthermore, the integration of base editing with transcriptomic profiling offers a systematic approach for interrogating the downstream effects of cancer driver mutations at a global gene expression level. Future applications of this platform in patient-derived organoids and multi-omics analyses could provide deeper insights into tumor biology and mutation-specific therapeutic vulnerabilities. Overall, our findings reinforce the potential of base editing as a powerful tool for dissecting cancer driver mutations and highlight its translational promise in precision oncology. By providing a scalable and versatile strategy to functionally assess oncogenic alterations, this work contributes to the broader effort of developing mutation-targeting therapeutic interventions.

## Methods

### gRNA design

sgRNAs were manually designed to match the mutant sequence in question and subsequently assessed with the following algorithms: DeepSpCas9 [123] to assess Cas9 binding, DeepBE [124] and BEdeepon [125] for assessing adenine base editing efficiencies as well as CasOFFinder [126] to find potential off-targets.

### gRNAs and primers used

**Table:** 

gRNA	Sequence
TP53-R273H	GTGCATGTTTGTGCCTGTCC
TP53-R175H	GAGGCACTGCCCCCACCATG
KRAS-G12D	GCTGATGGCGTAGGCAAGAG
SMAD4-Q311*	AGGCTAGAATGCAAGCTCAT
PTEN-R233*	CCGTCATGTGGGTCCTGAAT
VEGFA3	GGTGAGTGAGTGTGTGCGTG
Nonsense gRNA	CCTCCAGTTCATGCCGCCCA

**Table:** 

Primer	Sequence
TP53-R273H fwd	GTGCTAGGAAAGAGGCAAGGA
TP53-R273H rev	CTGCTTGCCACAGGTCTCC
TP53-R175H fwd	CAACCACCCTTAACCCCTCC
TP53-R175H rev	CGCCAACTCTCTCTAGCTCG
KRAS-G12D fwd	TGGACCCTGACATACTCCCA
KRAS-G12D rev	AGCGTCGATGGAGGAGTTTG
SMAD4-Q311* fwd	AGTTCTTAGACATTGCATAAGCTTGT
SMAD4-Q311* rev	TCCAGTTAACCAGAGATCCTGA
PTEN-R233* fwd	TGCCACTAGAAGTCTAATTTTGGGA
PTEN-R233* rev	TCACCAATGCCAGAGTAAGCA
VEGFA3 fwd	GTGCAGACGGCAGTCACTAGG
VEGFA3 rev	TATTGGAATCCTGGAGTGACCC

### Plasmids

For gRNA cloning into the lentiviral plasmid, protospacers were cloned into the LRT2B vector expressing tdTomato (Addgene plasmid #110854), using BsmBI/BbsI sites following the standard protocol. Unless a guanine was the first base in the protospacer, a guanine was added to the 5′ end of the protospacer before cloning to boost the expression of the gRNA from the human U6 promoter. Oligos for gRNA (containing cacc-/aaac-overhangs) were phosphorylated and annealed in a 20-µL reaction containing 100 pmol of each gRNA oligo (sense + antisense), 2 µL 10 × T4 ligation buffer and 0.5 µL T4 polynucleotide kinase. The reaction was run in a thermocycler at 37 °C for 30 min, 95 °C for 5 min, and then ramped down to 25 °C at a rate of 0.1 °C/min. GoldenGate cloning was then performed in a 20-µL volume with: 1 µL of 1/100 diluted hybridized oligos, 60 ng backbone vector, 2 µL 10 × T4 ligation buffer, 1 µL BsmBI and 1 µL T4 ligase. The reaction was run in a thermocycler as follows: 6 × (37 °C for 5 min, 23 °C for 5 min) followed by incubation at 37 °C for 15 min, and finally, 80 °C for 5 min. Next, 2 µL of this final reaction was used to transform DH5a *E. coli* cells. A single colony was picked, grown in liquid LB-antibiotic media before plasmid DNA was purified (Thermo Fisher GeneJet DNA Miniprep Kit). The gRNA insert was verified with a forward primer to the U6 promoter: 5′-GAGGGCCTATTTCCCATGATTCC-3′. For the base editor cloning, the NG-ABE8e base editor was cloned into a lentiviral vector as previously described [37] and will be available from Addgene (Addgene plasmid #242000). Single clones were picked, grown, and miniprepped followed by sequencing employing several primers aligning the full sequence of the insert in addition to diagnostic test digests confirming the correct integration into the backbone. Finally, miniprepped plasmids were transformed into E. coli DH5a and cells were grown overnight at 37 °C with constant shaking. Plasmid maxiprep kits (Qiagen) were used to provide transfection-level DNA in a concentration of ~ 1 µg/µL. All plasmids were once again validated by Sanger sequencing and then used for lentivirus production (see also Additional file 2: Fig. S10).

### Lentivirus production and transduction

Lentiviral particle production was performed as follows: 7 million HEK293T (LentiX) cells were seeded in 10-cm dishes and transfected on the next day at 80% confluency with 2 µg VSV-G (pMD2.G, Addgene plasmid #12,259), 6 µg psPAX2 (Addgene plasmid #12,260;) and 10 µg of the transfer vector (e.g., pLenti-EF1a.NGABE8e-P2A-GFP-PGK-PuroR or pLenti-U6-EF1a-tdTomato-P2A-BlasR) using 35 µg PEI (1 mg/mL) per dish. After ~ 20 h of transfection, the medium was changed to complete DMEM and 72 h after transfection the viral supernatant was collected, filtered through a 0.45 µm filter, and centrifuged for 2 h at 50,000 g at 4 °C. The supernatant was decanted, and the viral pellets were resuspended in PBS overnight at 4 °C on a shaker. For long-term storage, the virus particles were kept in cryovials at − 80 °C. Alternatively (for some gRNA viruses), the viral supernatant was concentrated using Amicon Ultra-15 Centrifugal Filter Devices (100 kDa, Merck) according to the manufacturer’s instructions. When a new cell line was used for the first time, the amount of virus needed to infect 50% of the cells was determined by titration. Transductions were typically performed in 96-well plates in the presence of protamine sulfate (final concentration 5 µg/mL; Sigma-Aldrich) and spin-infected for 1 h, 1000 g at 37 °C.

### Cell culture

A431 (CRL-1555), HT-29 (HTB-38), PANC-1 (CRL-1469), NCI-H1975 (CRL-5908), NCI-H1155 (CRL-5818), and HEK293T (CRL-11268) cells were obtained and authenticated through ATCC. ESO-51 cells (ACC 694) were purchased and authenticated through the German Collection of Microorganisms and Cell Cultures (DSMZ), and HuCC-T1 cells (RCB-1960) were purchased and authenticated through the Riken Institute. The cell lines were maintained at 37 °C, 5% CO_2_ in the following media, with each supplemented with 10% (v/v) FBS (Gibco) and antibiotics (100 U/mL penicillin, 100 mg/mL streptomycin; Gibco), referred to henceforth as complete media:

**Table:** 

**HEK293T**	**DMEM** (high glucose, GlutaMAX, pyruvate)
A431	**DMEM** (high glucose, GlutaMAX, pyruvate)
HT-29	**McCoy's 5a** (high glucose, L-glutamine, Bacto-Pepton)
PANC-1	**DMEM** (high glucose, GlutaMAX, pyruvate)
NCI-H1975	**RPMI 1640** (+ L-glutamine)
ESO-51	**RPMI 1640** (+ L-glutamine)
HuCC-T1	**RPMI 1640** (+ L-glutamine)
NCI-H1155	**RPMI 1640** (+ L-glutamine, only 5% FBS)

Cell lines were routinely tested and confirmed to be Mycoplasma-free (latest on September 19, 2024). For all cell lines used, cells were allowed to recover after thawing for two passages before performing experiments.

### Flow cytometry

All cell lines were typically transduced in 96-well plates, and the percentage of infected cells was analyzed by measuring GFP/tdTomato expression using a MACSQuant VYB Analyzer (Miltenyi Biotec). At 72 h after transduction, cells were trypsinized and collected for flow cytometry analysis. Viable single cells were gated using the forward and side scatter, followed by doublets exclusion (see example gating below). GFP fluorescence was measured using a blue 488 nm laser, and tdTomato was measured using a 561 nm yellow laser. Log area of the signal was collected. For GFP/tdTomato gating, a gate was defined using the appropriate wildtype so that ~ 1% positive signal remained, which was later subtracted (Additional file 2: Fig. S11). Cell sorting was carried out using a BD FACSMelody™ Cell Sorter (BD Biosciences, NJ, USA).

For time course experiments, starting day 3, adherent biological triplicates were measured every 3–6 days (dependent on cell growth). For each time point, medium was carefully washed with 200 µL sterile PBS and treated with 25–30 µL/well Trypsin–EDTA (0.25%, Gibco), just enough to cover the adherent layer of cells. Cells were incubated for 5–10 min at 37 °C. Next, 170 µL complete medium was added directly to the cells. The cell suspension was homogenized by pipetting up and down vigorously. Then, 20–50 µL were transferred to a new 96-well plate for later acquisitions and filled up to 200 µL with prewarmed complete medium. Of the remaining 170 µL cell solution, 50–100 µL were used for flow cytometry. For DNA isolation at day 3, the remaining cells from all replicates of each condition were pooled and used for gDNA extraction.

### Genotyping of base edited cells

Genomic DNA was isolated using the QIAamp DNA Blood Mini Kit according to the manufacturer’s instructions. Targeted PCR amplification of the respective exons was performed using high-fidelity Phusion polymerase according to the manufacturer’s instructions. Briefly, for one 50-µL PCR reaction, 10 µL HF buffer was added to 200–300 ng genomic DNA and mixed with 1 µL dNTPs (10 mmol/L) in addition to 1.25 µL of each forward and reverse primer (20 mmol/L). Then, the reaction mix was brought to 49.5 µL using nuclease-free water, and 0.5 µL Phusion DNA polymerase was added. Reagents were mixed, briefly spun down at room temperature, and run in a thermocycler with the following cycling conditions:

• 30 s 98 °C (initial denaturation)
• 35 × :
  - ◦ 15 s 98 °C (denaturation).
  - ◦ 30 s 65 °C (annealing, specific to primer pairs).
  - ◦ 45 s 72 °C (extension).
• 5 min 72 °C (final extension).
• 8 °C (hold).

A 5-µL aliquot of each PCR reaction was run on agarose gel, confirming correctly sized bands and purity of procedure through a blank no-template water control. PCR products were purified using ISOLATE II PCR and Gel Kit (Bioline) according to the manufacturer’s instructions, and DNA concentrations were quantified using a Nanodrop spectrophotometer. The appropriate amount of DNA, together with the respective sequencing primers (forward + reverse primer were used), was submitted for Sanger sequencing, following the vendor’s protocol.

### EditR to quantify base editing efficiency

EditR [127] is a free online tool to quantify sequencing reads from raw ab1 files and the gRNA protospacer sequence (20 bp). For quantification of base editing efficiency, forward + reverse Sanger sequencing reactions of the target condition were averaged, and forward + reverse Sanger sequencing reactions of cells infected with the control gRNA were subtracted (= background). If editing was estimated from a mixed population (e.g., only 50% infected with gRNA virus), the resulting editing was normalized to the gRNA-expression level (e.g., divide by 0.5).

### Rechallenging residual ABE-gRNA expressing cells with mRNA or sgRNA

PANC-1-ABE-gRNA cells were kept in complete DMEM in addition to puromycin (2 µg/mL) and blasticidin (20 µg/mL). For transfections, 2 × 10^5^ cells were seeded in 24-well plates 1 day prior to transfections and transfected using in vivo-jetRNA + (Polyplus) transfection reagent according to the manufacturer’s recommendations. We used 100 pmol gRNA (R273H-targeting or *VEGFA3*-targeting, Synthego) or 2 pmol ABE mRNA (IVT mRNA, generated according to the manufacturer’s guidelines using the HiScribe T7 ARCA mRNA Kit (NEB, Ipswich, MA, USA)), and kept a ratio (w/v) of RNA:Transfection reagent at 1:2. Seventy-two hours post transfection, half the cells were collected and genomic DNA was isolated. Then, the *TP53*-R273H and *VEGFA3* loci were amplified using Phusion polymerase (NEB) followed by Sanger sequencing (Microsynth) to reveal the editing efficiency. The other half of the cells were kept in culture for two more days, and live cell counts were acquired using flow cytometry (MACSQuant VYB, Miltenyi Biotec, Bergisch-Gladbach, Germany) at 5 days post transfection.

### RNA-seq analysis

The raw data from the three cell lines were aligned to the human genome hg38 using STAR aligner v2.7.3a [128] after quality check with FastQC v0.12.1 [129]. The annotation used for mapping was GENCODE v46 [130] [B]. Consequently, using featureCounts v2.0.6 [131] a count matrix was generated for 42 samples of the R273H mutation for further downstream analysis. For the R175H mutation, the analysis was performed on 4 samples. All genes with no reads were discarded. Principal component analysis (PCA) was performed using genes with the highest variance. The differential expression analysis was performed using DESeq2 [132]. The fold change cutoff was set at 2 and *p*-value threshold was set at 0.05 for considering the differentially expressed genes. Heatmaps were clustered based on hierarchical clustering. Gene ontology analysis was performed with the goseq [133] algorithm.

#### Statistical analysis

Data was analyzed using Excel. Unless otherwise stated, time points in time-course experiments are presented as the SDs (presented as error bars) of three independent experiments, performed in biological triplicates. For base editing time courses, the raw FACS points were processed using FlowJo, and the statistical difference between the mean percentage at the end point of experimental gRNA and that of control gRNA/no gRNA was determined using an unpaired two-tailed Student *t* test. *p* < 0.05 was considered to be statistically significant.

## Supplementary Information

Additional file 1: Table S1. DEGs overlap in TP53-R273H corrected cell lines within cell lines and with cancer gene census

Additional file 2: Figure S1. Time courses after infection with gRNA-viruses showing percent tdTomato-expressing population on an absolute scale. Figure S2. Re-challenging residual PANC-1 cells after time course with ABE mRNA/gRNA. The remaining double positive cells are editable via transfection with chemically synthesized gRNAs. Figure S3. RT-qPCR for U6-gRNA expression in PANC1-ABE-GFP-gRNA-Tomato cells vs Freshly infected PANC-1-ABE-GFP cells. RT-qPCR for U6-gRNA expression in PANC1-ABE-GFP-gRNA-Tomato cells reveals U6 silencing. Figure S4. Editing on RNA level after correction of TP53-R273H and Fluorescence-gRNA expression and editing on DNA level over selected timepoints. Figure S5. Comparison of DE genes after TP53-R273H correction. Volcano plots of early time pointsin the A431, HT-29 and PANC-1 lines in addition to overlap of DE genes between the three lines and Principal component analysis. Figure S6. Heatmaps of DE genes overlapping with the 116 p53 core targets in three lines over time. Figure S7. ChiP-seq peaks of putative p53 targets. Figure S8. Editing on RNA level after correction of TP53-R175H and overlap with the p53 core targets. Figure S9. Evaluation of gRNAs and applicability of ABE system on common TP53 and KRAS mutations. Figure S10. Plasmid maps of NG-ABE8e and gRNA. Figure S11. Exemplary gating strategy for doublet exclusion

Additional file 3: Table S2. DEGs in individual cell lines following correction TP53-R273H and SMAD-Q311* and overlap with Fischer, 2017

Additional file 4: Table S3. DEGs in TP53-R175H as well as in comparison to TP53-R273H cell lines

## Data Availability

The full DeSeq2 result tables and source code from this study, particularly code used to analyze RNA-seq, can be found at the corresponding Zenodo Github page (https://zenodo.org/records/15640640) [134, 135]. RNA-seq datasets generated in this study can be found at the Gene Expression Omnibus (GEO) under GSE287868 [136].
