# Supplementary material for: A base editing platform for the correction of cancer driver mutations unmasks conserved p53 transcription programs
Source: Genome Biol. 2025 Jul 22;26:217. doi: 10.1186/s13059-025-03667-7 (PMC12285138; doi:10.1186/s13059-025-03667-7)
Supplement: Supplementary file 2 — Additional file 2: Figure S1. Time courses after infection with gRNA-viruses showing percent tdTomato-expressing population on an absolute scale. Figure S2. Re-challenging residual PANC-1 cells after time course with ABE mRNA/gRNA. The remaining double positive cells are editable via transfection with chemically synthesized gRNAs. Figure S3. RT-qPCR for U6-gRNA expression in PANC1-ABE-GFP-gRNA-Tomato cells vs Freshly infected PANC-1-ABE-GFP cells. RT-qPCR for U6-gRNA expression in PANC1-ABE-GFP-gRNA-Tomato cells reveals U6 silencing. Figure S4. Editing on RNA level after correction of TP53-R273H and Fluorescence-gRNA expression and editing on DNA level over selected timepoints. Figure S5. Comparison of DE genes after TP53-R273H correction. Volcano plots of early time pointsin the A431, HT-29 and PANC-1 lines in addition to overlap of DE genes between the three lines and Principal component analysis. Figure S6. Heatmaps of DE genes overlapping with the 116 p53 core targets in three lines over time. Figure S7. ChiP-seq peaks of putative p53 targets. Figure S8. Editing on RNA level after correction of TP53-R175H and overlap with the p53 core targets. Figure S9. Evaluation of gRNAs and applicability of ABE system on common TP53 and KRAS mutations. Figure S10. Plasmid maps of NG-ABE8e and gRNA. Figure S11. Exemplary gating strategy for doublet exclusion [file 13059_2025_3667_MOESM2_ESM.docx]

**A base editing platform for the correction of cancer driver mutations unmasks conserved p53 transcription programs**

Pascal Wang^1^, Rituparno Sen^1^, Frank Buchholz^1,2,3,4^, and Shady Sayed^1^

^1^Medical Systems Biology, Medical Faculty, Technical University Dresden, Dresden, Germany.

^2^National Center for Tumor Diseases (NCT), Dresden, Germany.

^3^German Cancer Research Center (DKFZ), Heidelberg, Germany.

^4^German Cancer Consortium (DKTK), Dresden, Germany.

Correspondence: Frank Buchholz ([frank.buchholz@tu-dresden.de](mailto:frank.buchholz@tu-dresden.de), ORCID: 0000-0002-4577-3344) or Shady Sayed ([shady.sayed@tu-dresden.de](mailto:shady.sayed@tu-dresden.de), ORCID: 0000-0002-3209-238X).

### **Additional Files**

### **Additional file 2: Figure S1: Time courses after infection with gRNA-viruses showing percent tdTomato (gRNA)-expressing population on an absolute scale.**


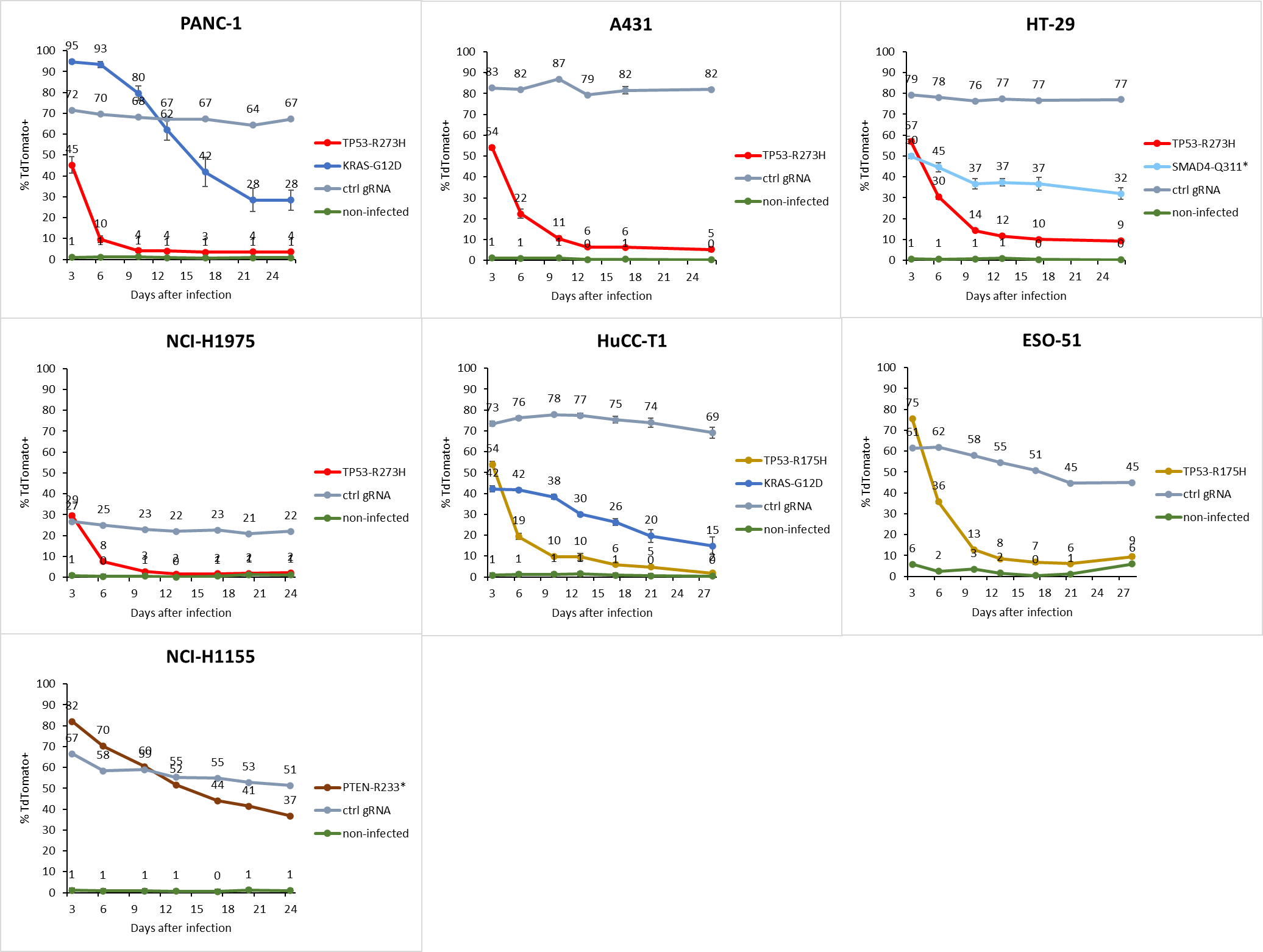


### **Additional file 2: Figure S2: Re-challenging residual PANC-1 cells after time course with ABE mRNA / gRNA.**


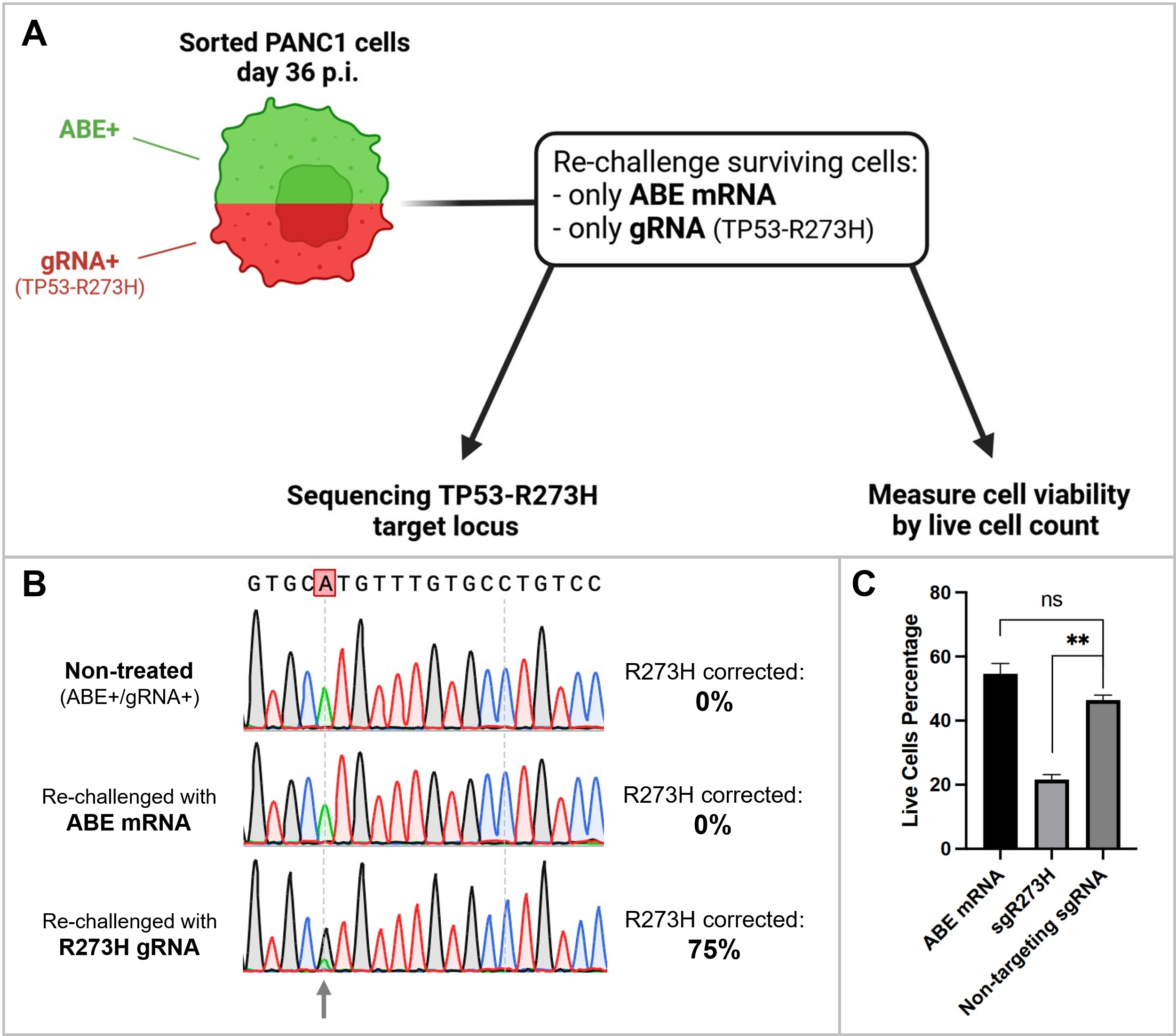

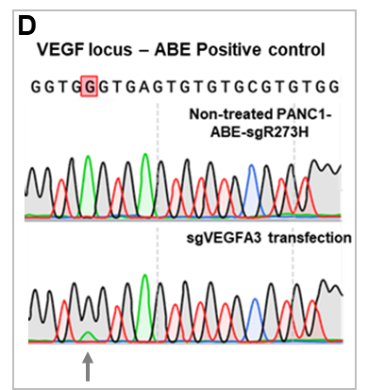


**Fig. S2: Remaining double positive cells are editable via transfection with chemically synthesized gRNAs.** (A) Experimental scheme representing PANC-1 cells stably expressing both ABE-GFP and R273H-gRNA-tdTomato rechallenged with either ABE mRNA or R273H-mutation-targeting gRNA. (B) Representative Sanger sequencing chromatograms highlighting the R273H locus (arrow) in PANC-1-ABE-gR273H cells treated with indicated conditions. Note the efficient A-to-G conversion (75%) in the cells re-challenged with the R273H-gRNA. (C) Cell viability measurement (shown as live cell population) day 5 following indicated treatments. Note the >50% viability deficit when cells were treated with the R273H gRNA. Error bars represent mean ± SD from biological duplicates performed in two independent experiments. Statistical significance was assessed using unpaired student’s t-test comparing mean live cells percentage of ABE-mRNA sample to that of sgR273H. ** indicates p<0.005. (D) VEGF gRNA transfection in PANC-1-ABE-gR273H cells. Representative Sanger sequencing chromatograms highlighting VEGFA3 locus in non-treated PANC-1-ABE-gR273H cells (top) with target A highlighted by an arrow, or after treatment with VEGFA3 synthetic gRNA (bottom). Cells transfected by only VEGFA3-gRNA reveals editing indicated by overlapping A/G peaks, confirming ABE expression in these cells.

### **Additional file 2: Figure S3: RT-qPCR for U6-gRNA expression in PANC1-ABE-GFP-gRNA-Tomato cells vs Freshly infected PANC-1-ABE-GFP cells**


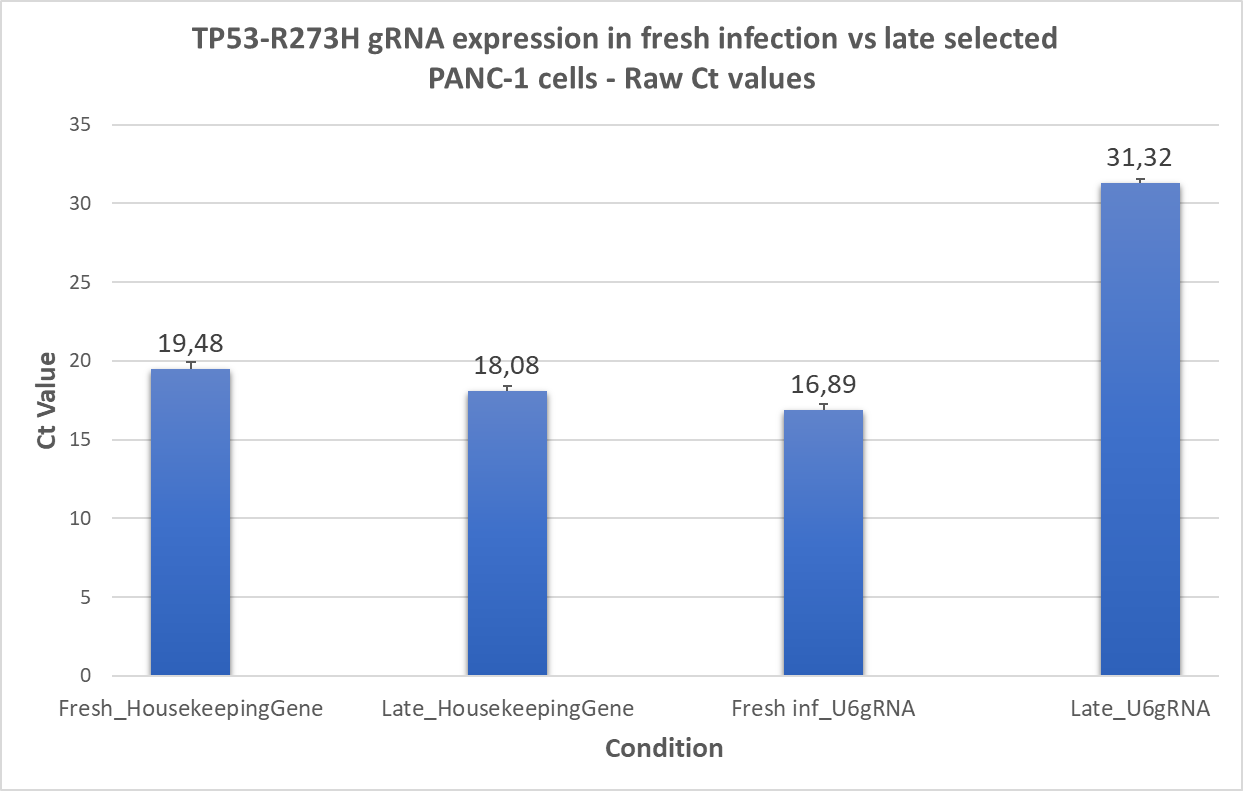

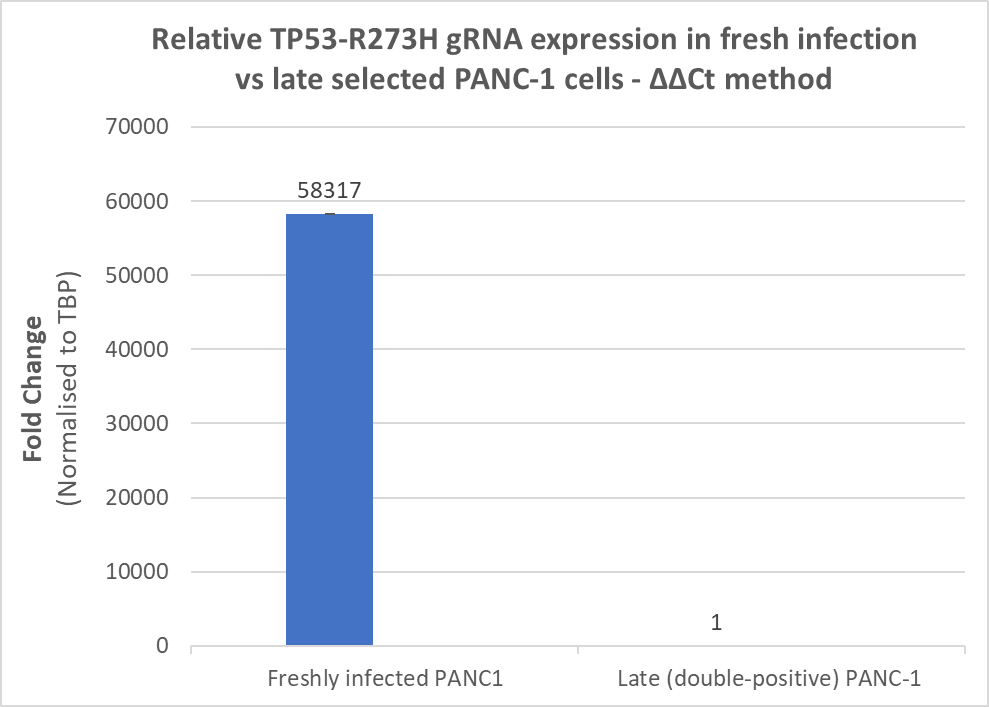


**Fig. S3: RT-qPCR for U6-gRNA expression in PANC-1-ABE-GFP-gRNA-Tomato cells reveals U6 silencing.** RT-qPCR utilizing random hexamers using the R273H-gRNA spacer as a forward primer, with the reverse primer in antisense orientation on the tracrRNA while using TBP as housekeeping gene on the plate. PANC-1-ABE cells that were freshly infected with the TP53 gRNA virus were used as a control for gRNA expression. Results show ~14 Ct cycles difference (ΔΔCt = 15.8) in the expression of gRNA in fresh infection vs PANC1-ABE-GFP-gRNA-Tomato cells, which translates to a >58000-fold difference (2^15.8^ = 58317).

### **Additional file 2: Figure S4: Additional information relating to RNA-seq after *TP53*-R273H correction.**

**A** Editing on RNA level after correction of *TP53*-R273H


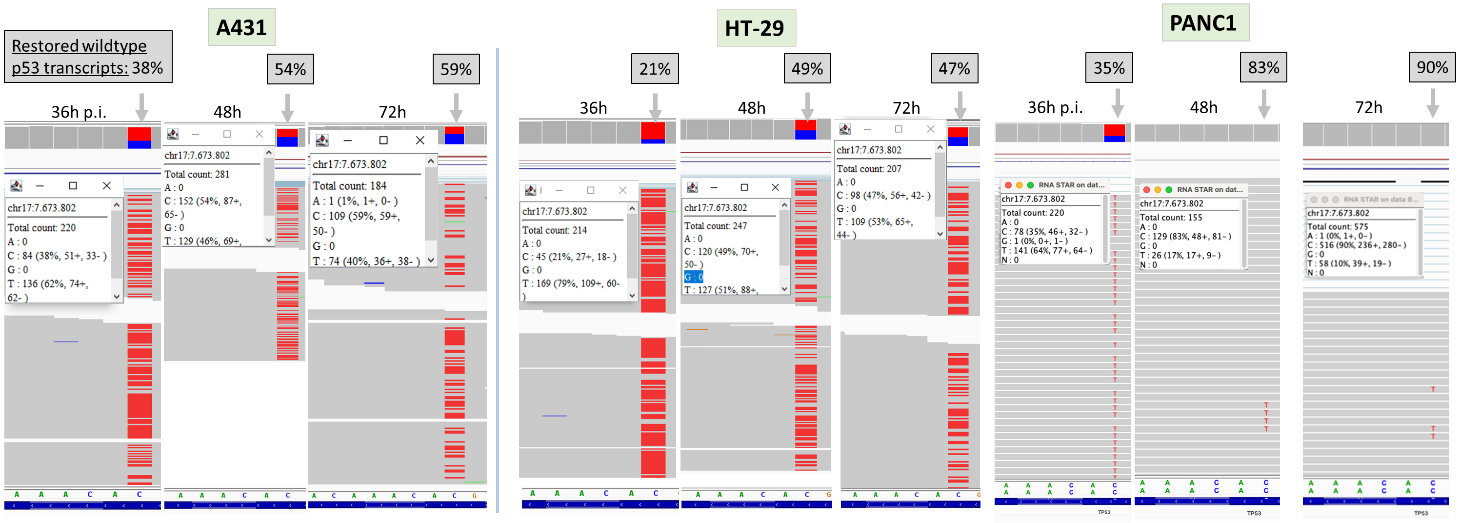


**B** (Fluorescence-gRNA expression and **DNA** editing) over selected timepoints


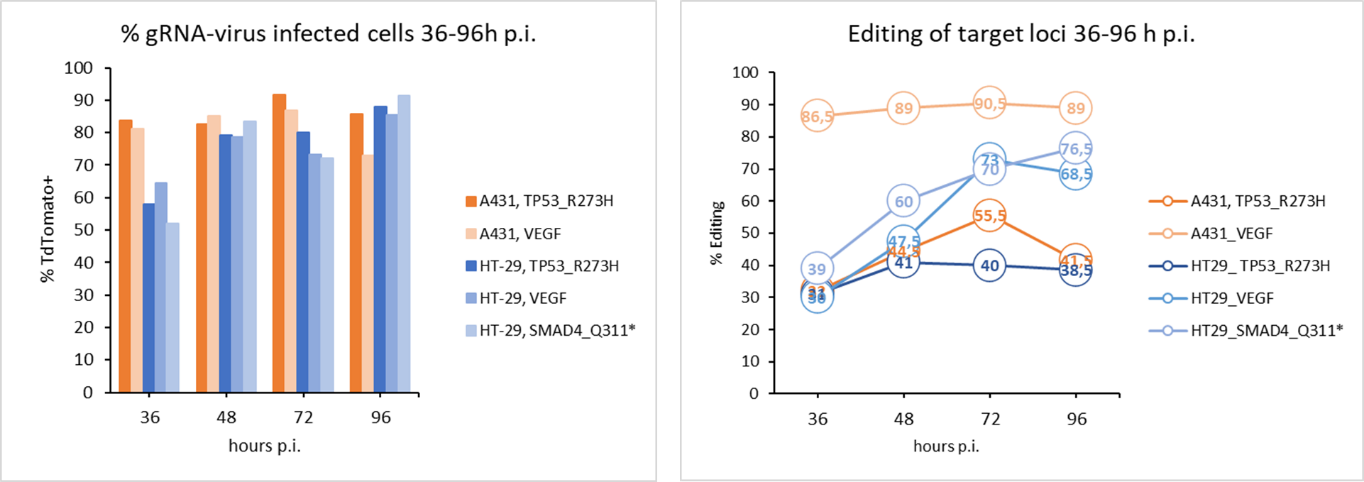


***Fig. S4.*** *(A) Editing on RNA level in A431, HT-29 and PANC-1 lines after correction of* TP53*-R273H 36h-72h post infection. Editing was measured as percent of corrected transcripts, as detected by RNA-seq. Results are displayed in IGV genome browser. (B) Percent tdTomato_gRNA expression of A431 and HT-29 line 36-96 hours after infection with R273H gRNA lentivirus (indicating gRNA expression) (Left) and total DNA editing of the bulk population (Right).*

### **Additional file 2: Figure S5: Comparison of DE genes after *TP53*-R273H correction.**

**(A) Volcano plots of early time points (36h + 48hpi) in the A431, HT-29 and PANC-1 lines.**


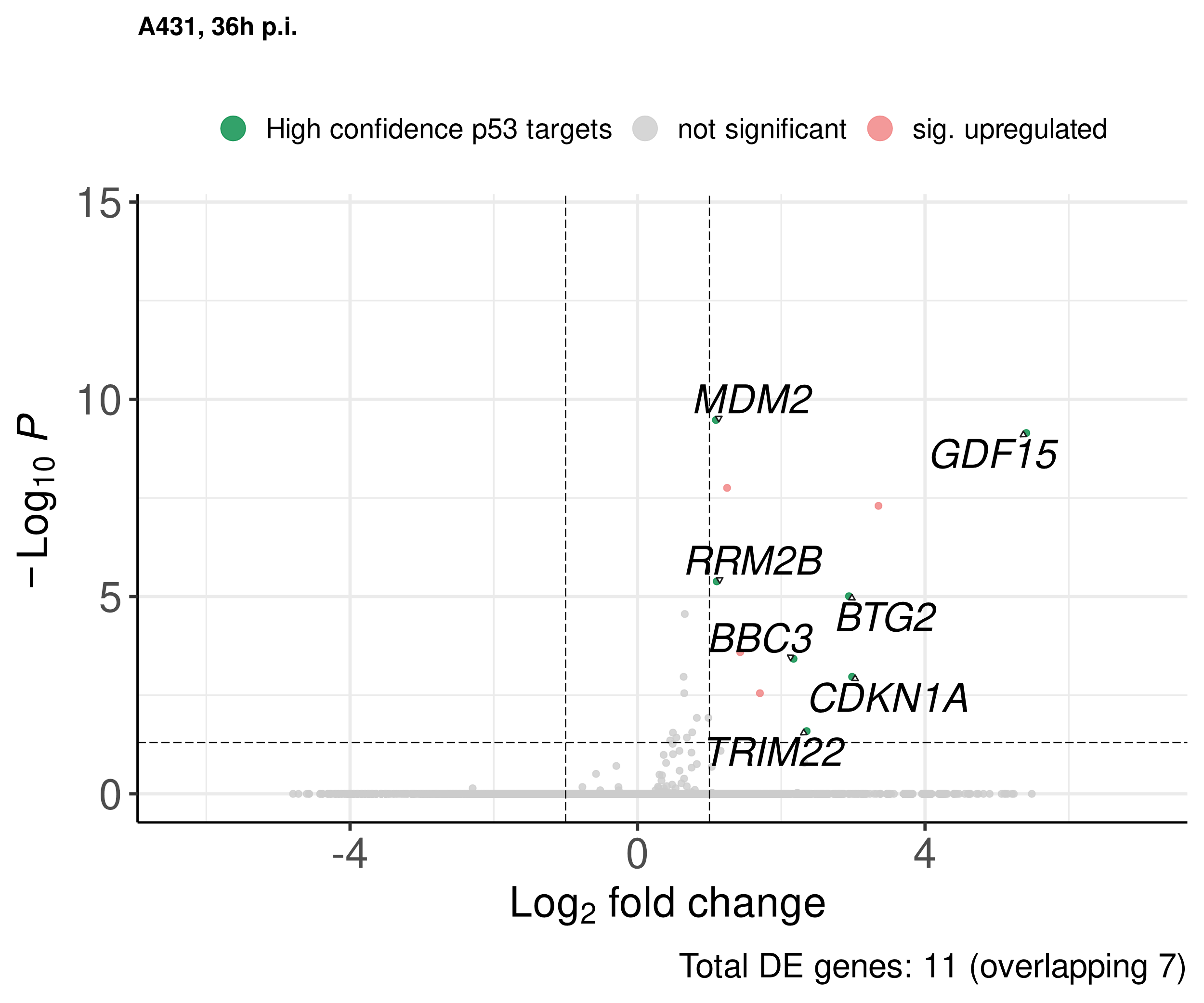

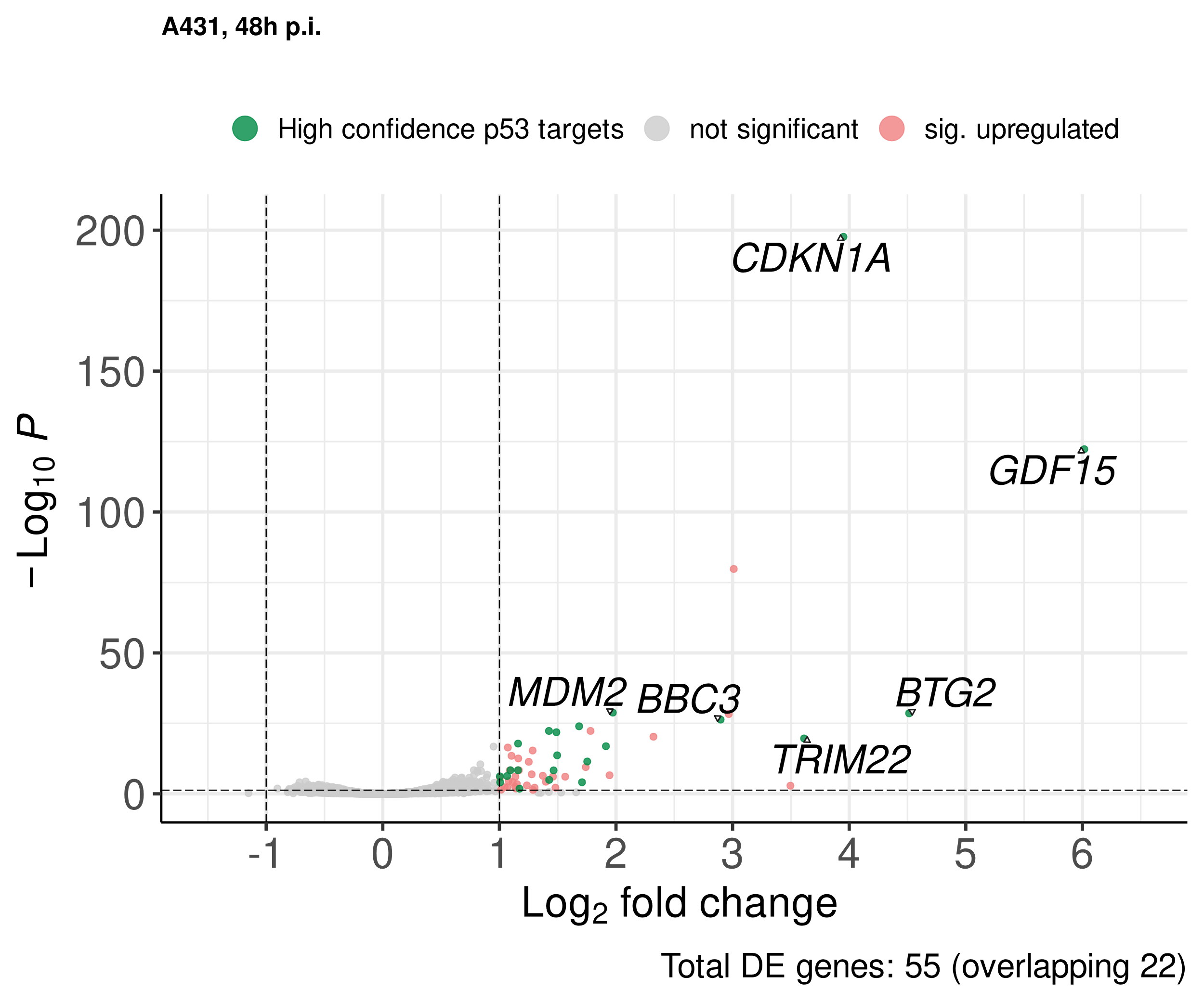

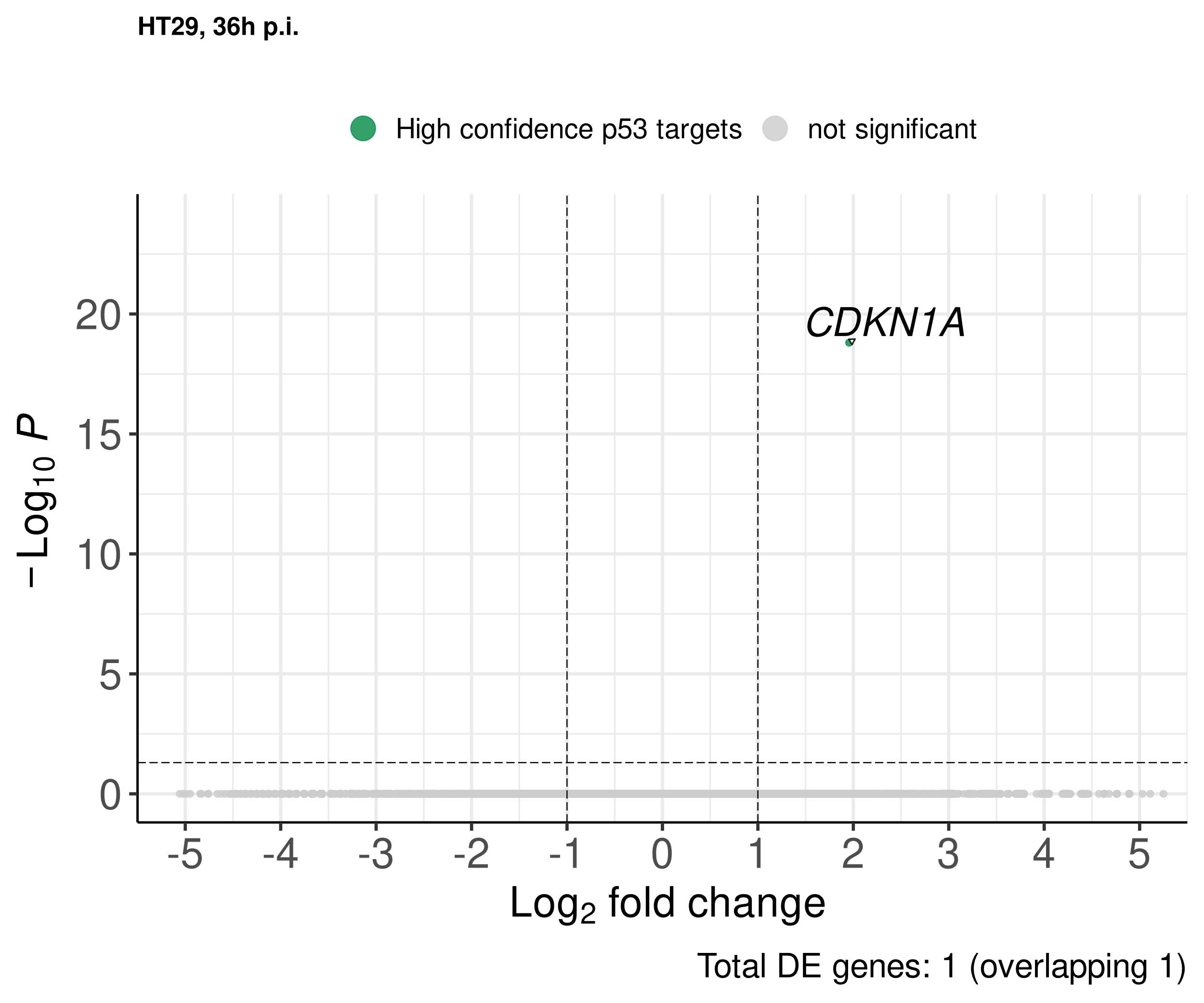

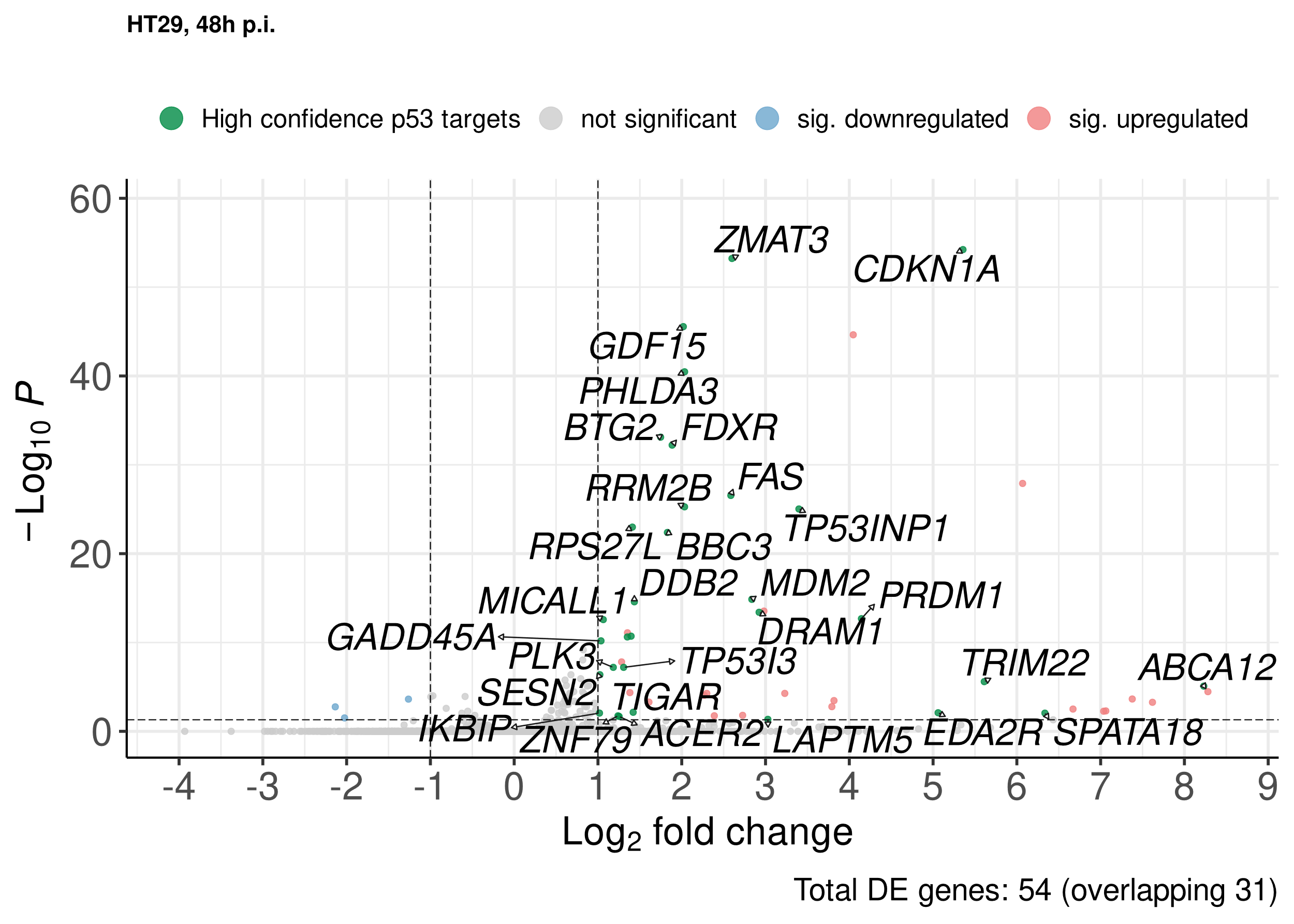

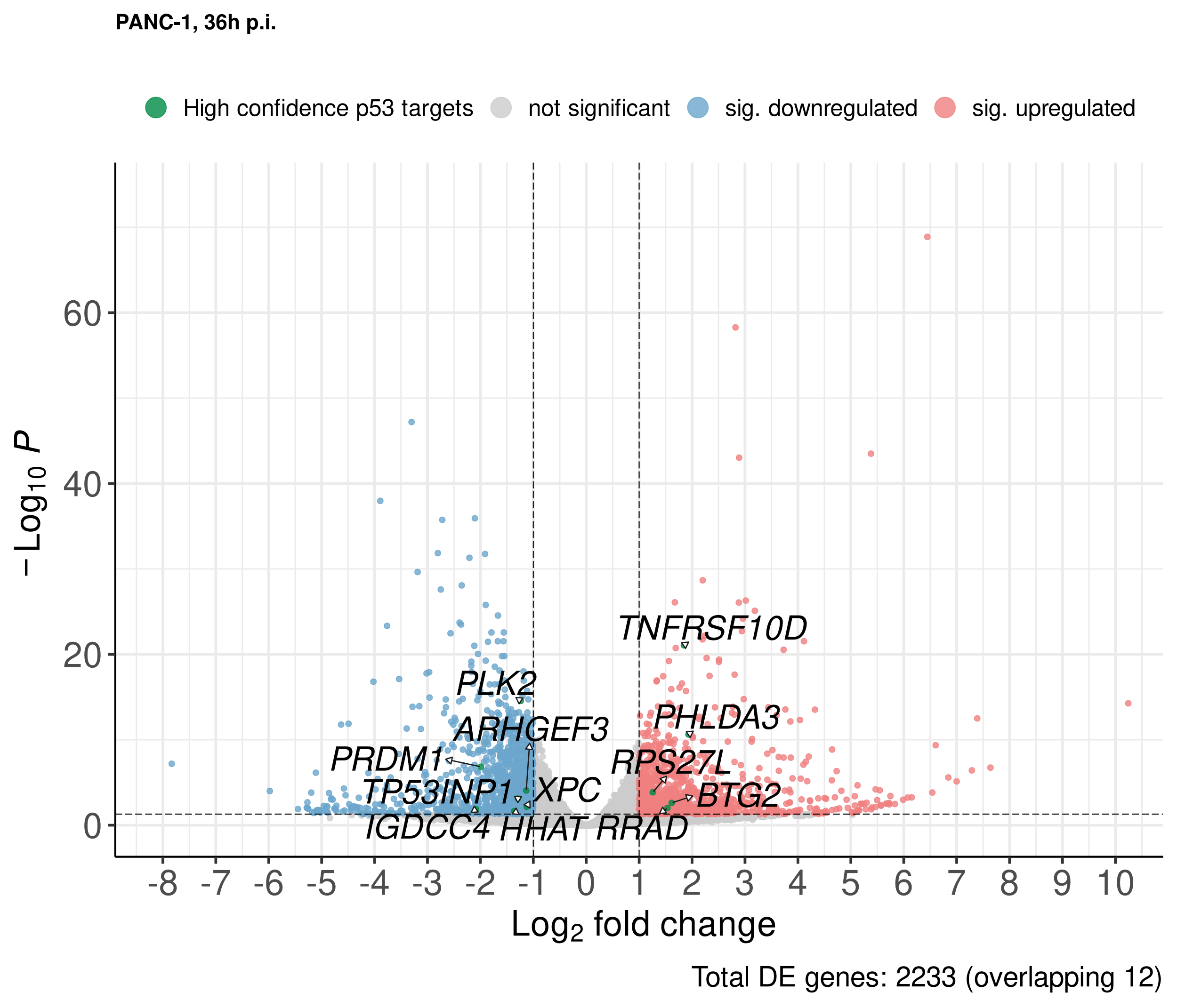

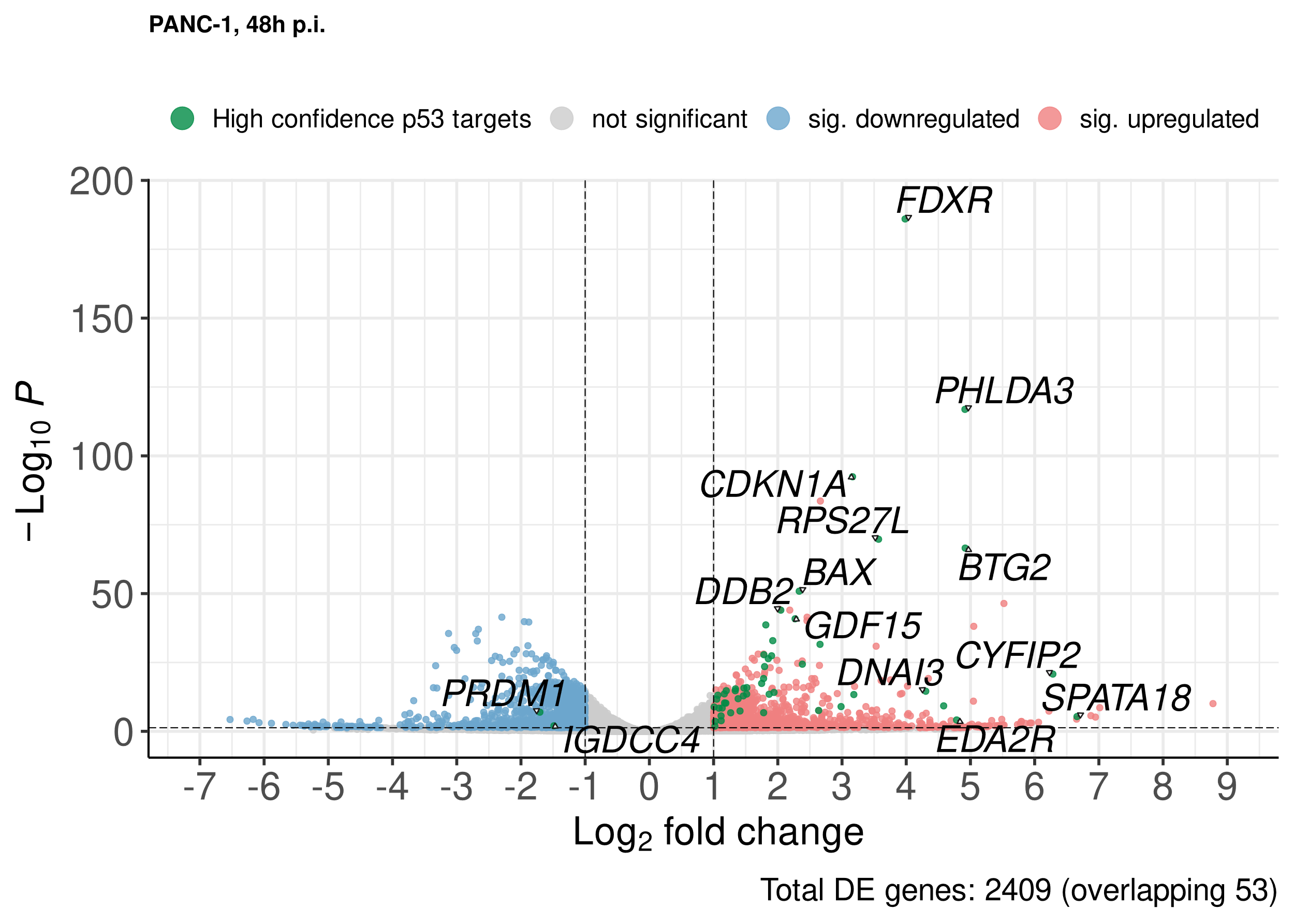


**(B) Left: Overlap of DE genes between the three lines, (C) Right: Principal component analysis (PCA)** of all analyzed replicates showing clustering within the cell lines + Venn diagram of overlapping mutations reported in PANC-1, A431 and HT-29 as reported in the depmap database.


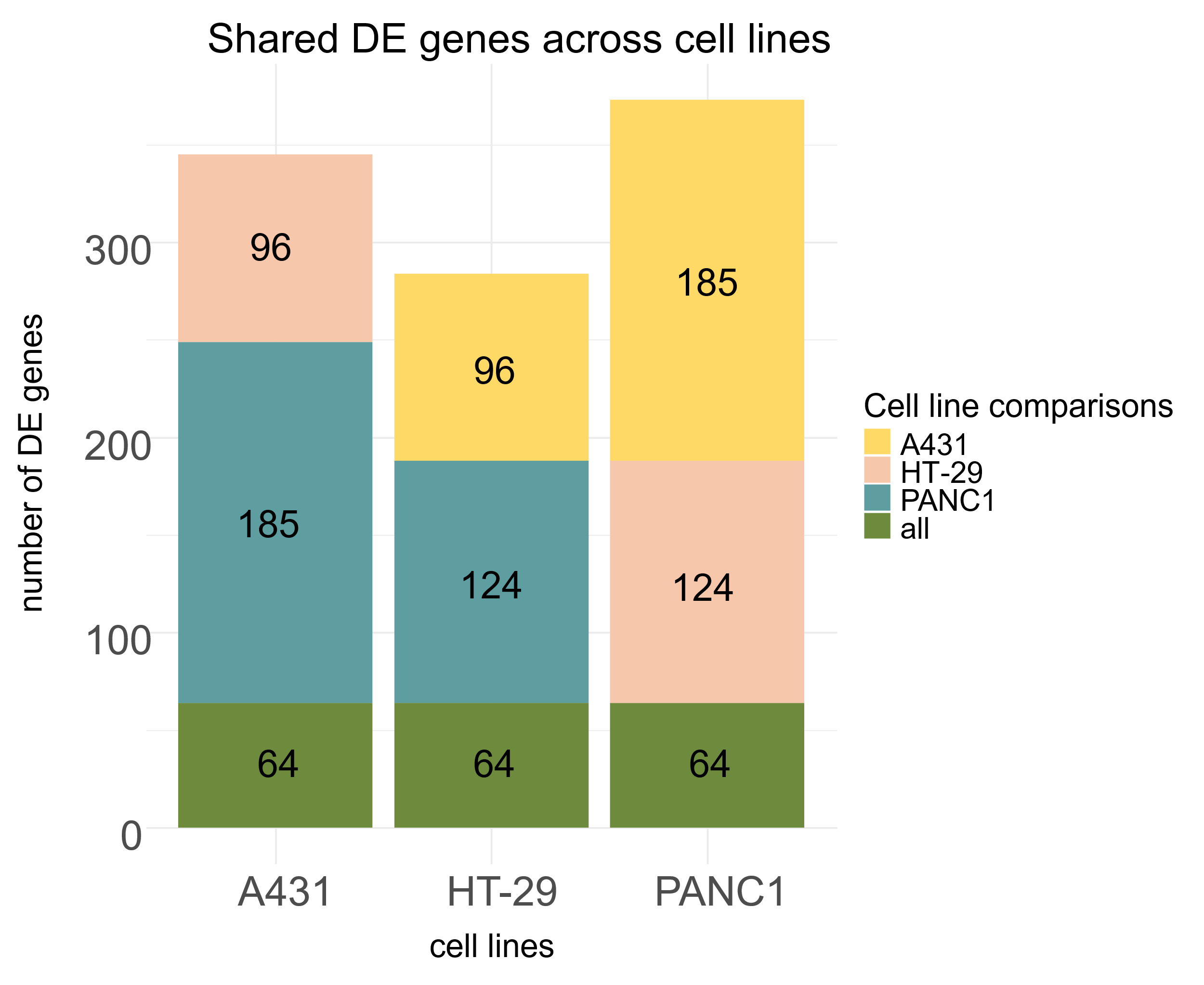

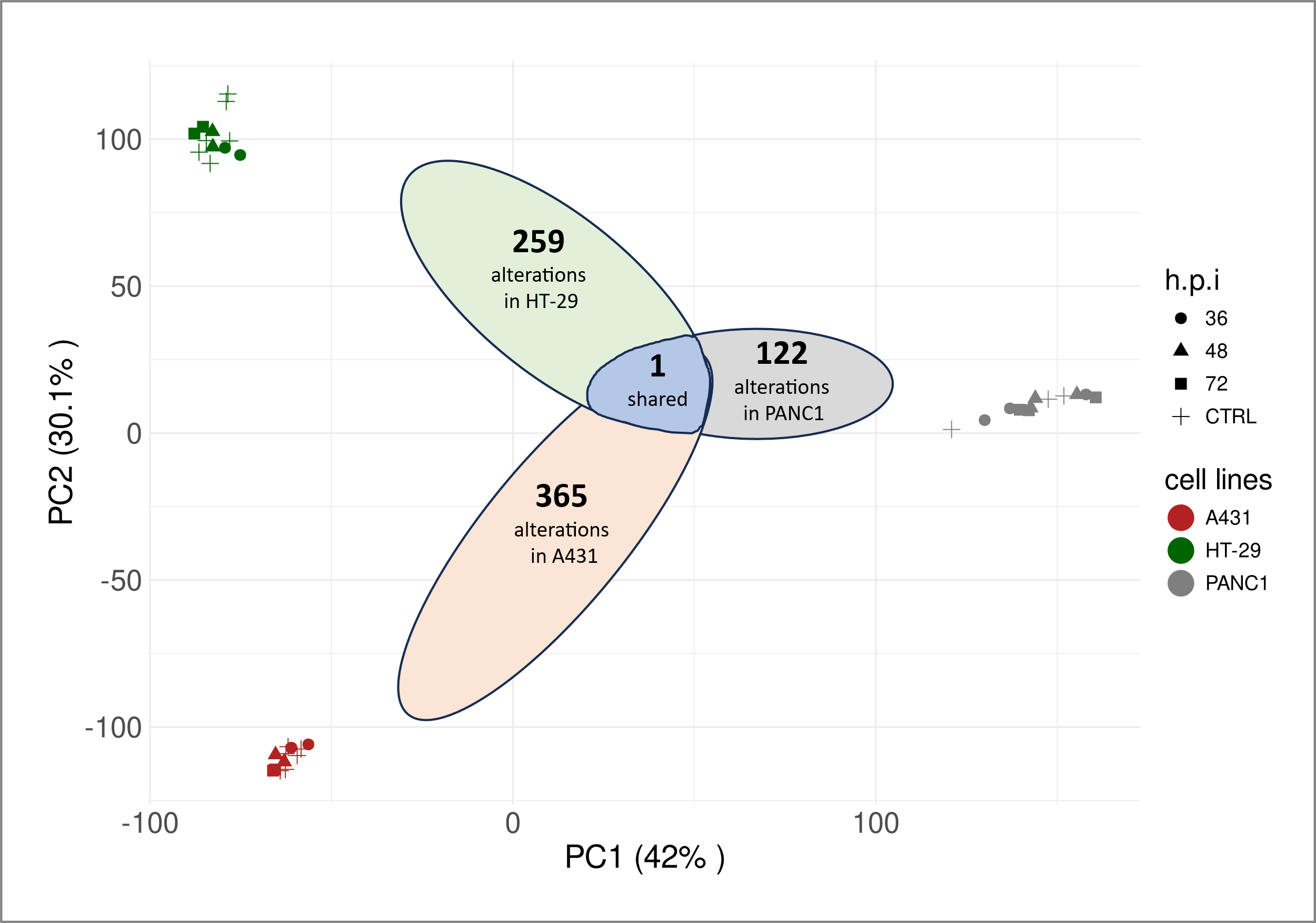


### **Additional file 2: Figure S6: Core p53 targets in the three lines over time.**


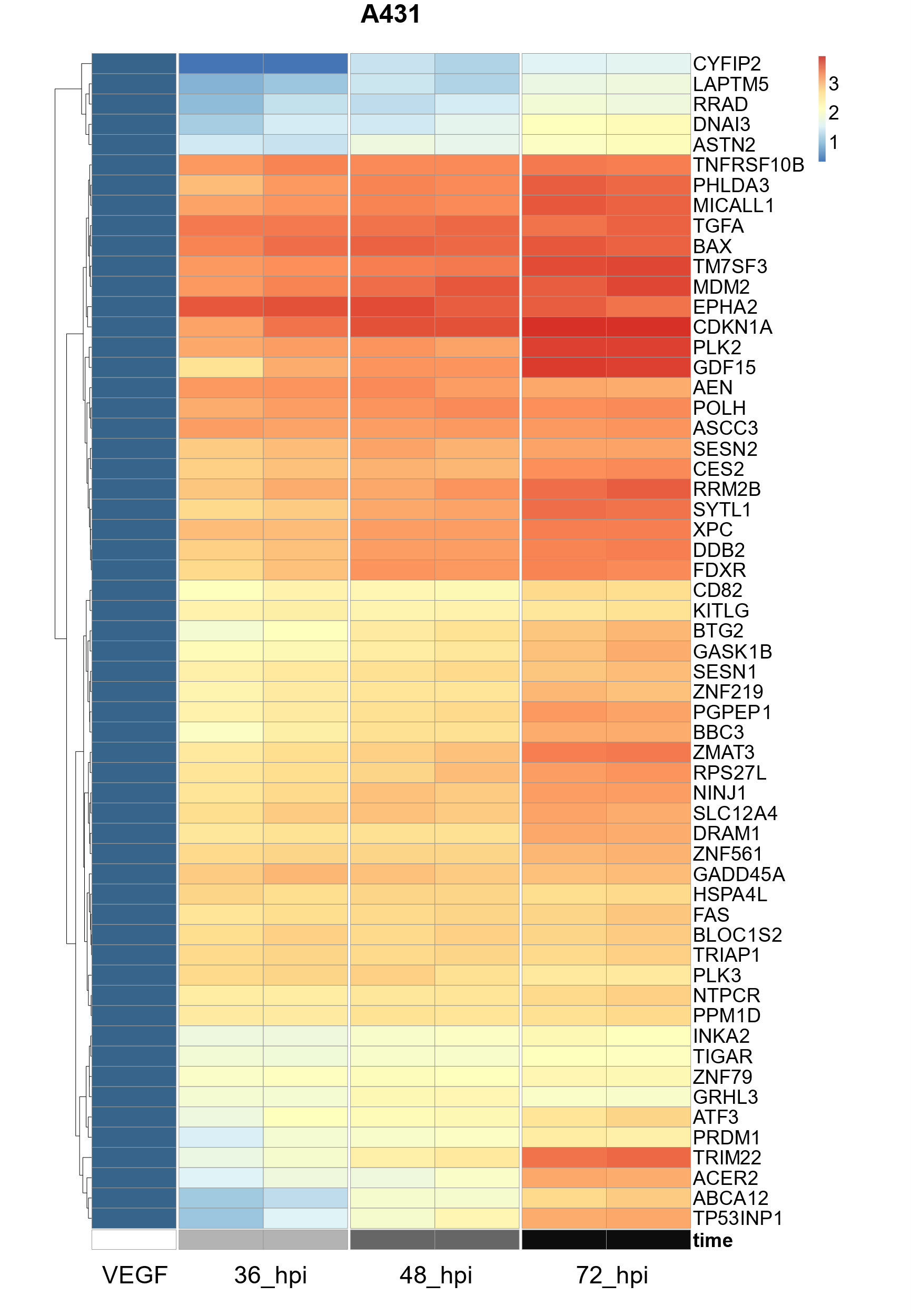

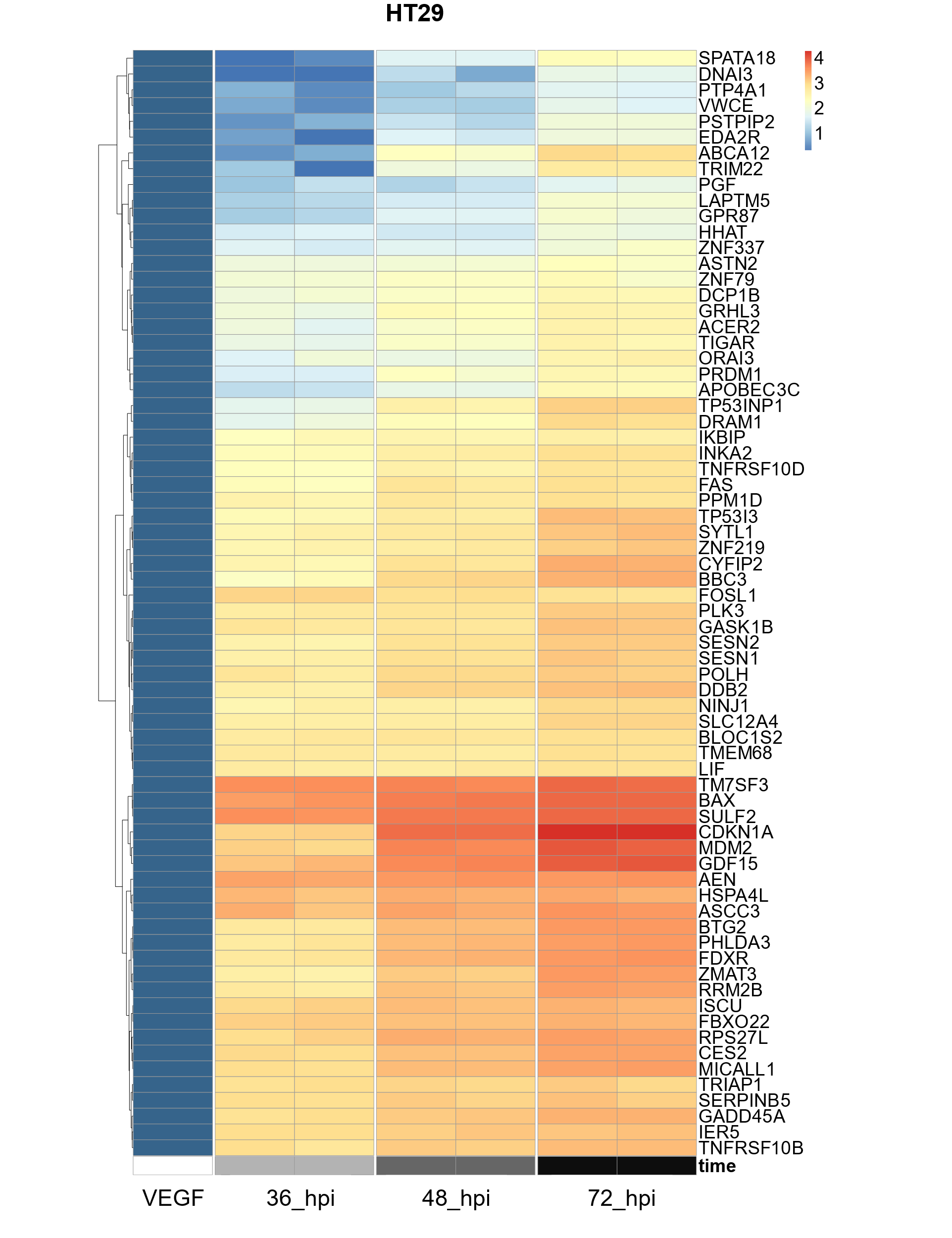

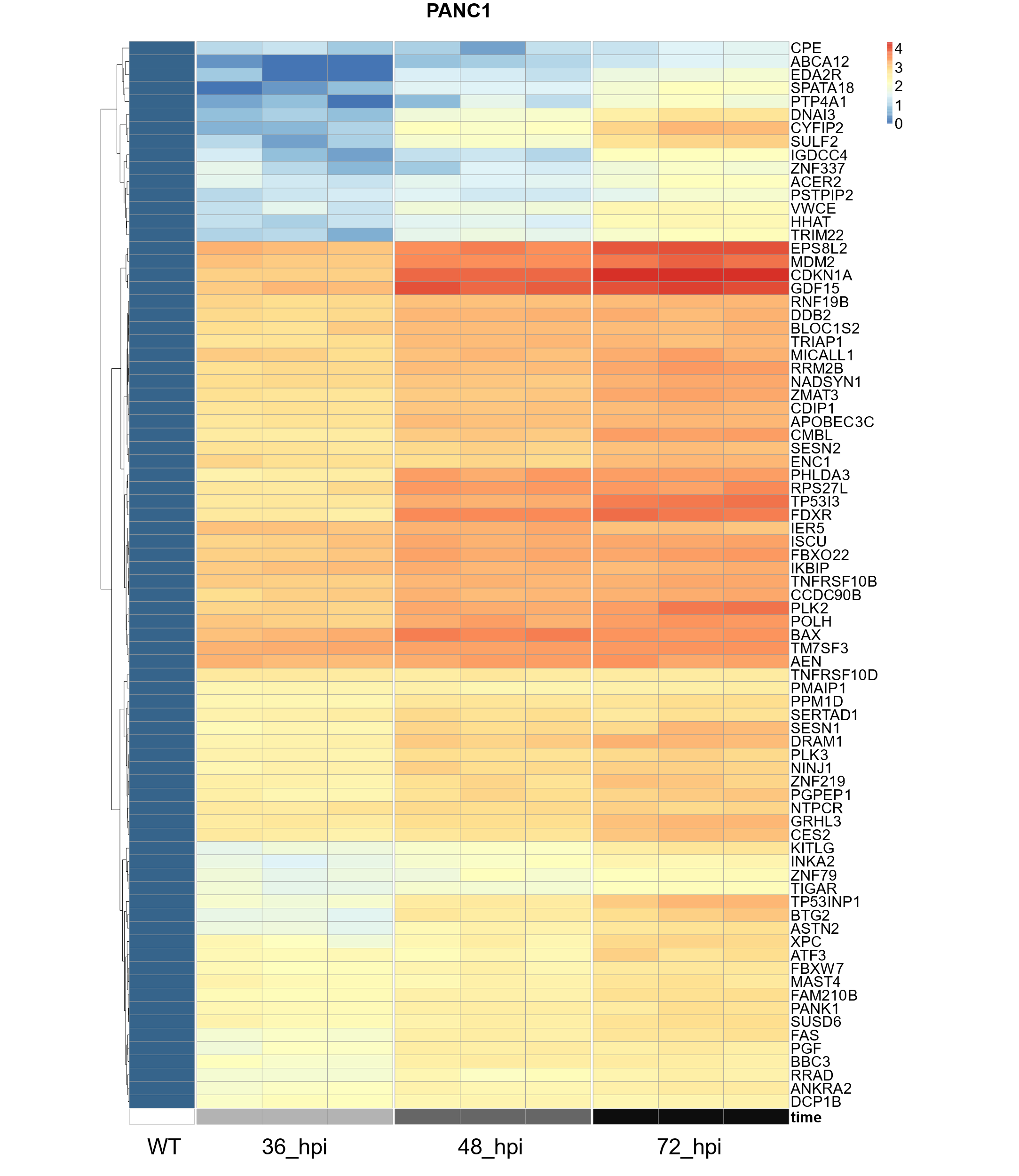


***Fig. S6: Heatmaps of DE genes overlapping with the 116 p53 core targets*** *from Fischer, 2017, 36-72hpi, grouped using hierarchical clustering, in all three lines.*

### **Additional file 2: Figure S7: ChiP-seq peaks of putative p53 targets.**


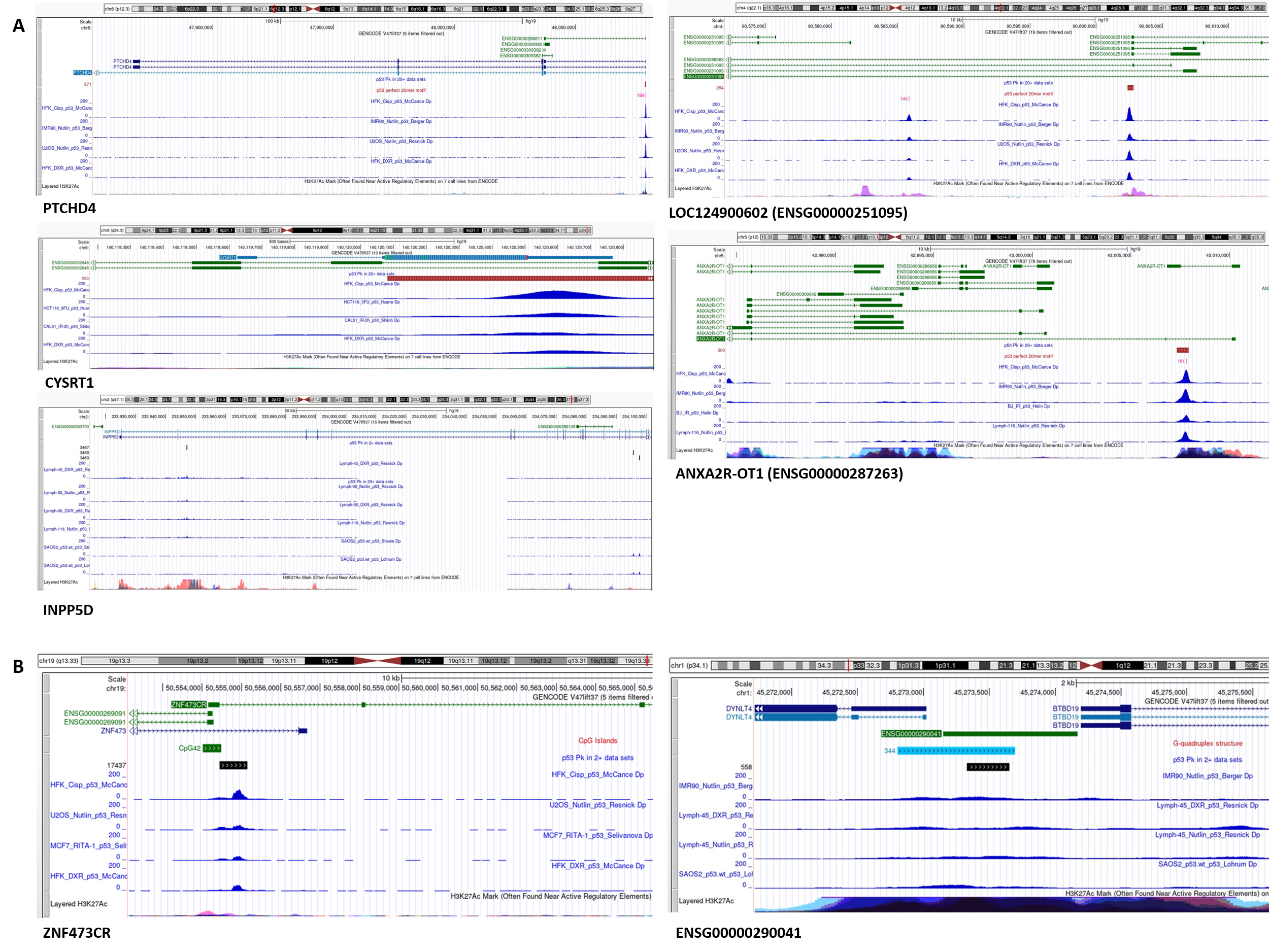


***Fig. S7. ChIP-seq peaks of putative p53 target genes.*** *Representative targets shown. The p53 UCSC BAER track*[68] *was used to identify peaks. (A) Transcripts being upregulated in all three lines (left) or in two out of three lines, and containing a p53 response element (right). (B) Two novel, undescribed lncRNAs, upregulated in two of three lines.*

### **Additional file 2: Figure S8: Additional information relating to RNA-seq following *TP53*-R175H correction**


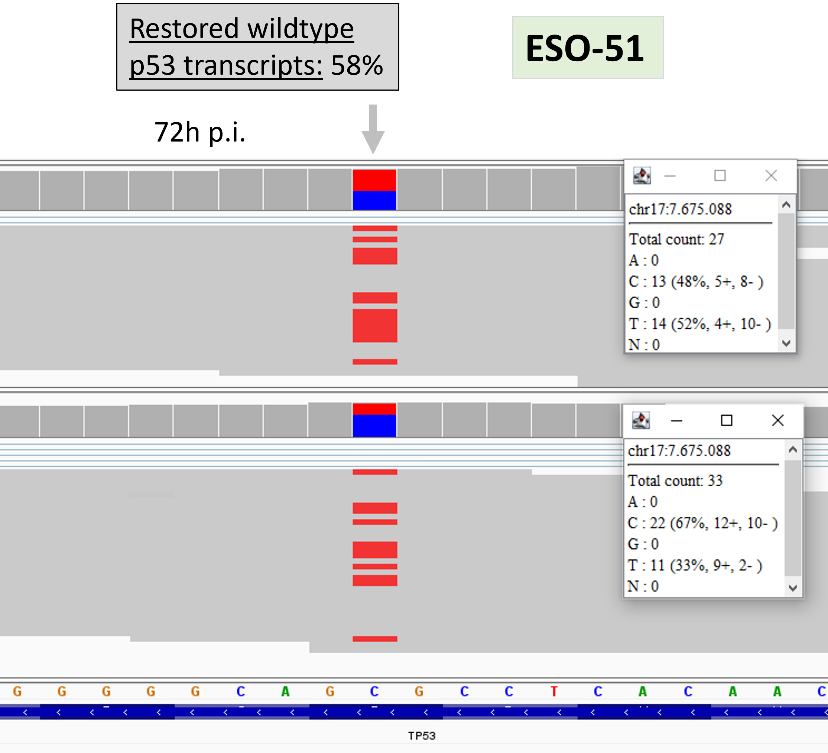
**A**

**B**

***Fig. S8. (A)*** *Editing on RNA level in ESO-51 line after correction of* TP53*-R175H 72h post infection in two replicates. Editing was measured as percent of corrected transcripts, as detected by RNA-seq. Results are displayed in IGV genome browser.* ***(B) Left:*** *High confidence p53 target genes are highlighted in orange if they overlap with either [62],[65] or [66]. Genes that overlap with ChIP-seq peaks from the UCSC p53 BAER track [65] are shown in bold.* ***Right:*** *Downregulated genes that belong to* *p53-p21-DREAM targets are highlighted in blue. See also: Additional file 4: Table S3.*

### **Additional file 2: Figure S9: Evaluation of gRNAs and applicability of ABE system on common *TP53* and *KRAS* mutations.**

***
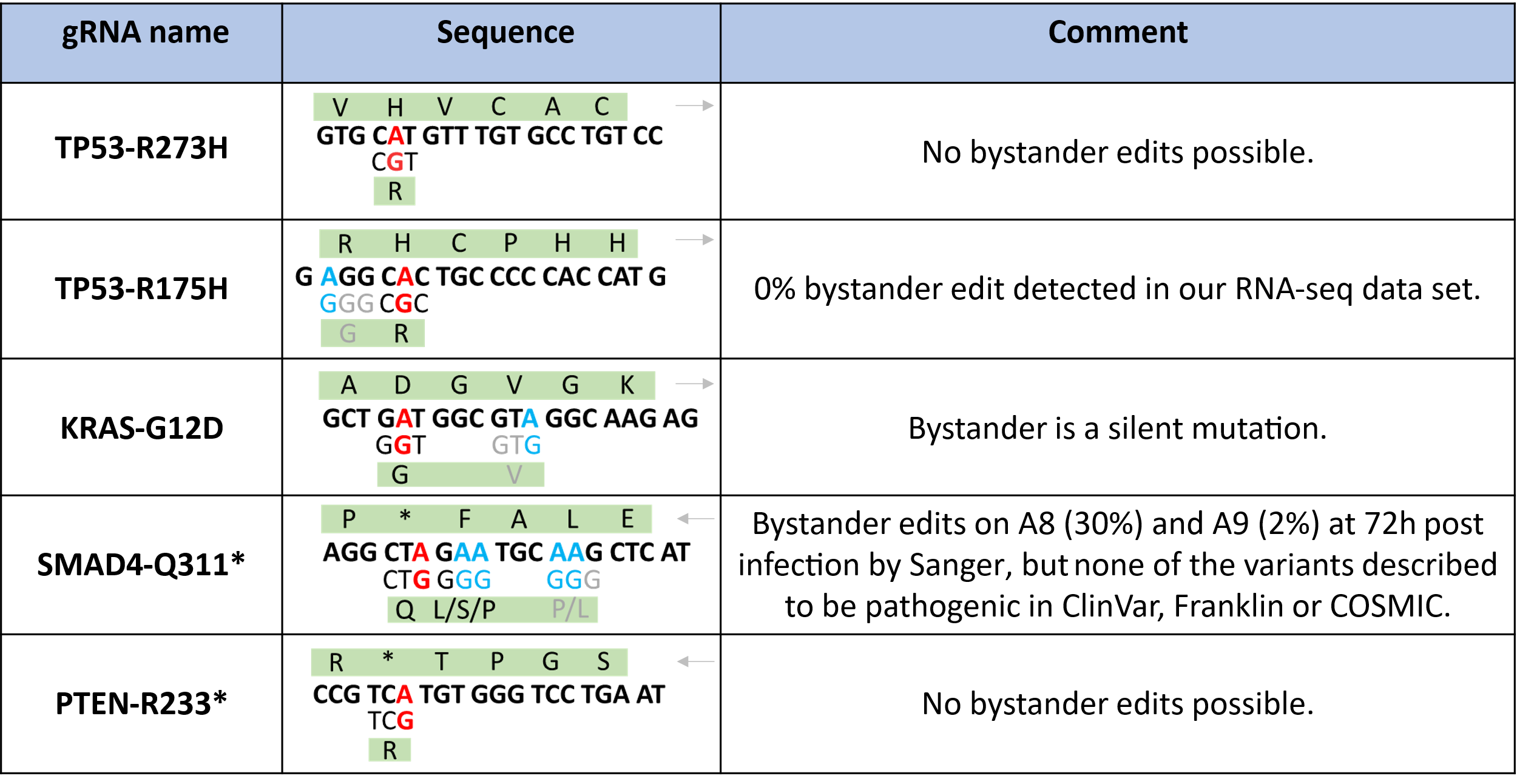
***

***Fig. S9a. Evaluation of gRNAs used in this study.*** *For each mutation, the gRNA sequence is depicted with Target A highlighted in red and Bystander A’s highlighted in blue. The corresponding amino acid is shaded in green.*


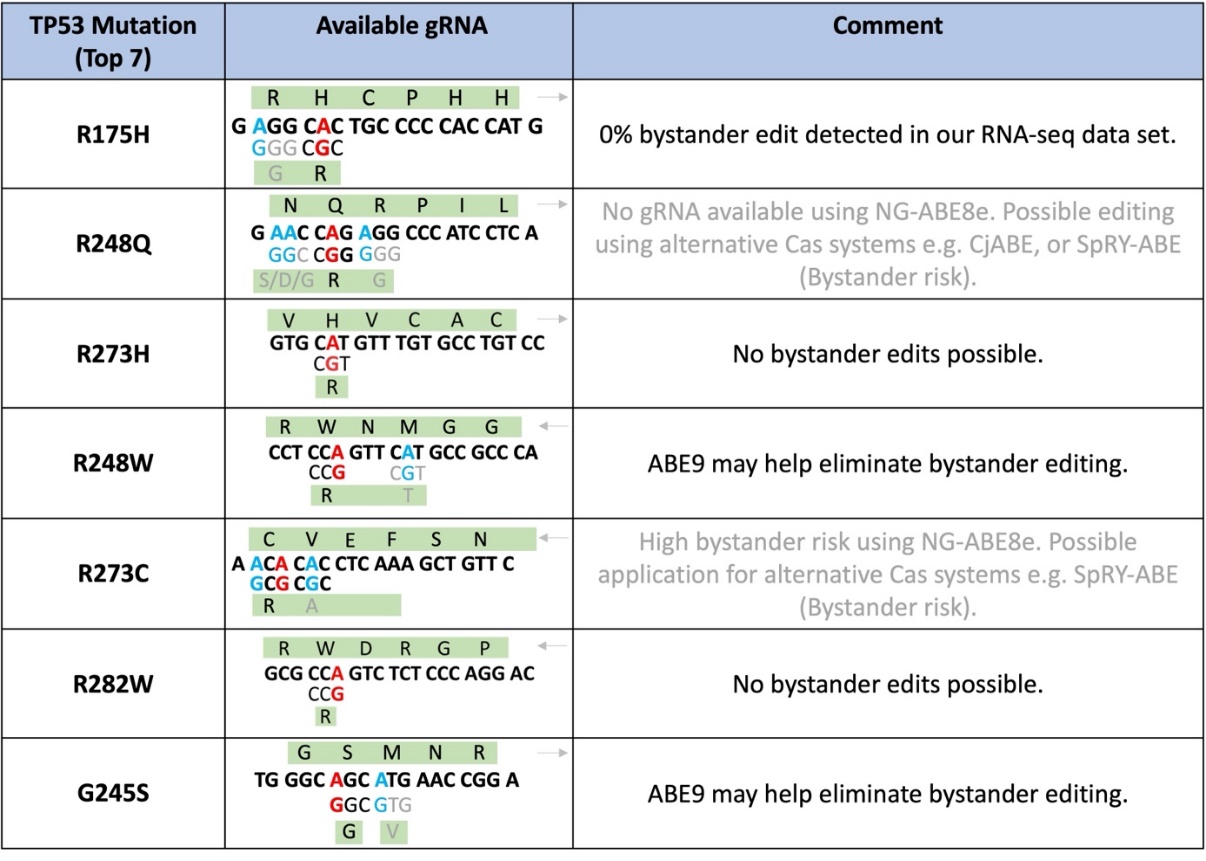


***Fig. S9b.*** *Potential gRNAs for the top 7 most common* TP53 *mutations (ranked by occurrence*[19]*). gRNAs are designed for the NG-ABE8e base editor, editing might still be possible with other systems (e.g. NG-ABE9, SpRY-ABE9).*


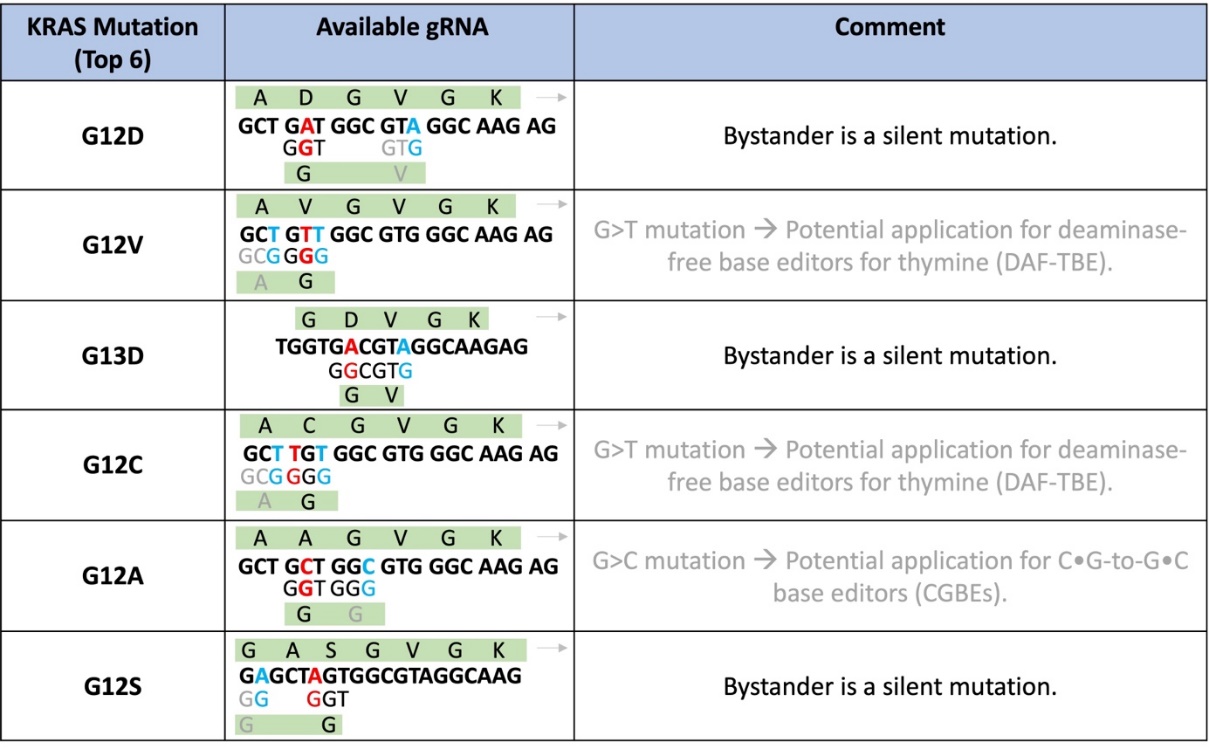


***Fig. S9c.*** *Potential gRNAs for the top 6 most common* KRAS *mutations (ranked by occurrence*[19]*). gRNAs are designed for the NG-ABE8e base editor, editing might still be possible with other systems (e.g. NG-ABE9, SpRY-ABE9).*

### **Additional file 2: Figure S10: Plasmid maps of NG-ABE8e and gRNA**


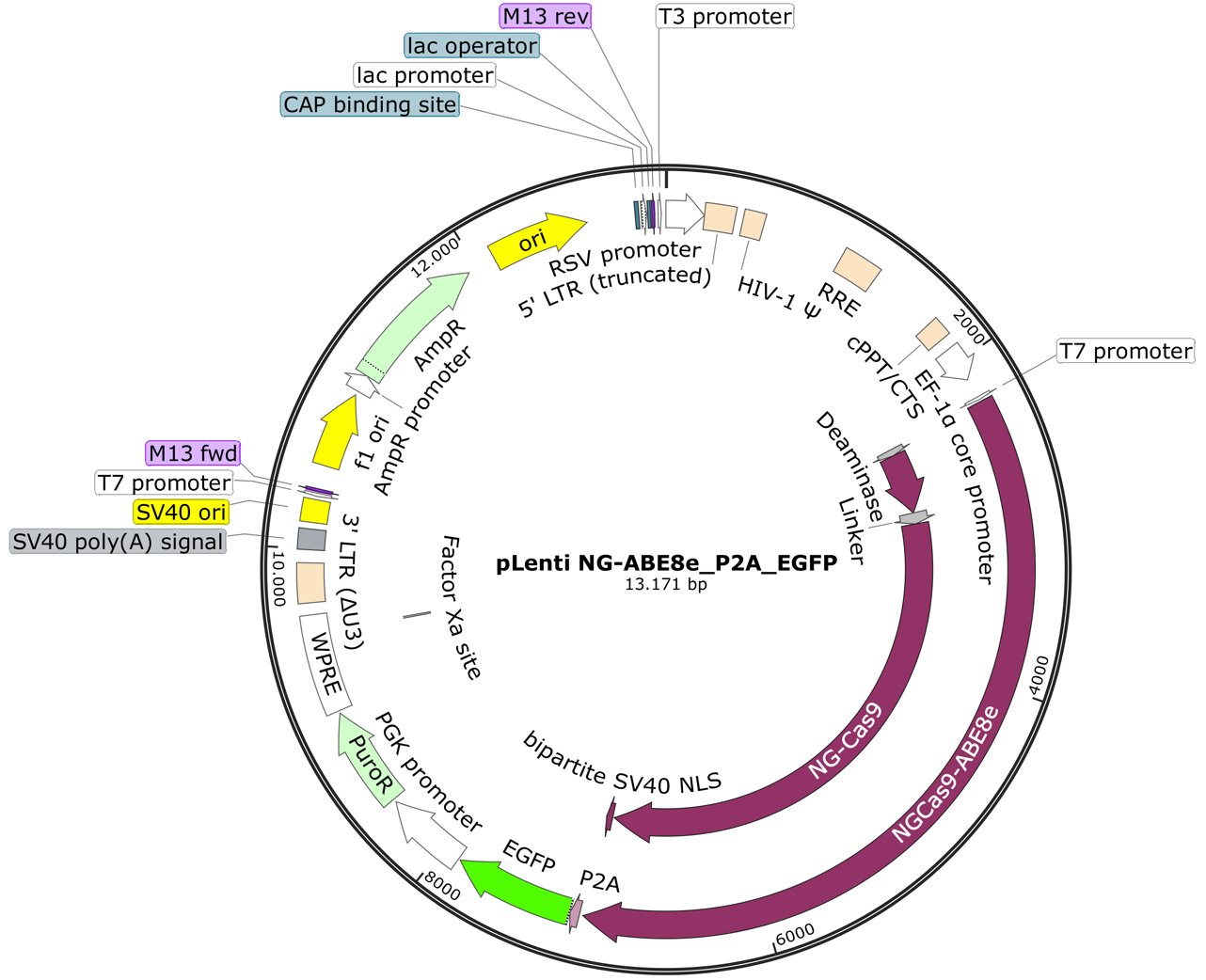

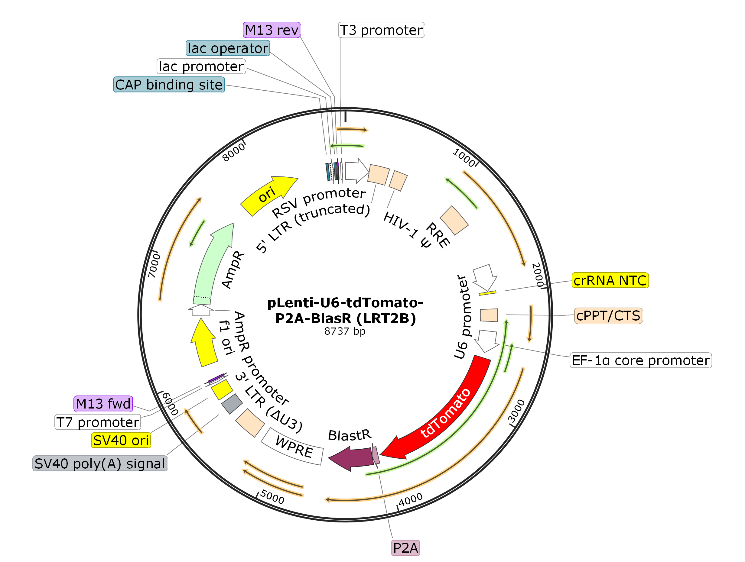


***Fig. S10. Plasmid maps of NG-ABE8e and gRNA vector plasmids used for lentivirus production.*** *NG-ABE8e is coupled to EGFP via a P2A site and contains a puromycin resistance gene (left). The gRNA plasmid contains a tdTomato and a blasticidin resistance gene (right).*

### **Additional file 2: Figure S11: Example gating for doublet exclusion**


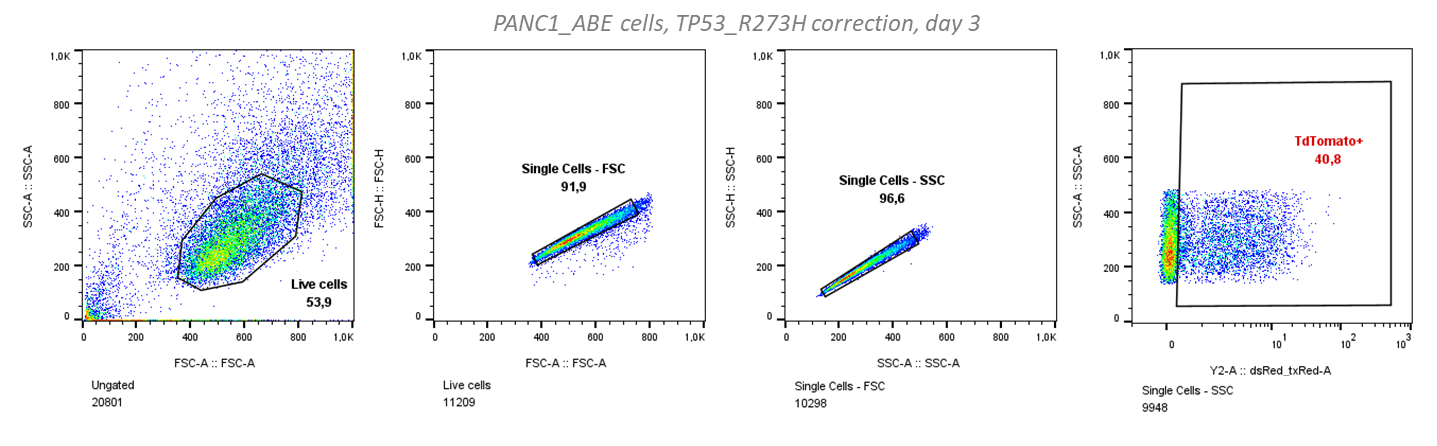
 ***Fig. S11.*** *Example gating of PANC1 cells, day 3 after infection with gRNA_tdTomato virus*
